# Regulation of PaRBOH1-mediated ROS production in Norway spruce by Ca^2+^ binding and phosphorylation

**DOI:** 10.3389/fpls.2022.978586

**Published:** 2022-10-13

**Authors:** Kaloian Nickolov, Adrien Gauthier, Kenji Hashimoto, Teresa Laitinen, Enni Väisänen, Tanja Paasela, Rabah Soliymani, Takamitsu Kurusu, Kristiina Himanen, Olga Blokhina, Kurt V. Fagerstedt, Soile Jokipii-Lukkari, Hannele Tuominen, Hely Häggman, Gunnar Wingsle, Teemu H. Teeri, Kazuyuki Kuchitsu, Anna Kärkönen

**Affiliations:** ^1^ Department of Agricultural Sciences, Viikki Plant Science Centre, University of Helsinki, Helsinki, Finland; ^2^ Department of Ecology and Genetics, University of Oulu, Oulu, Finland; ^3^ UniLaSalle, Agro-Ecology, Hydrogeochemistry, Environments & Resources, UP 2018.C101 of the Ministry in Charge of Agriculture (AGHYLE) Research Unit CS UP 2018.C101, Mont-Saint-Aignan, France; ^4^ Department of Applied Biological Science, Tokyo University of Science, Noda, Japan; ^5^ Organismal and Evolutionary Biology Research Programme, Faculty of Biological and Environmental Sciences, Viikki Plant Science Centre, University of Helsinki, Helsinki, Finland; ^6^ Natural Resources Institute Finland (Luke), Production Systems, Helsinki, Finland; ^7^ Meilahti Clinical Proteomics Core Facility, Biochemistry & Dev. Biology, University of Helsinki, Biomedicum-Helsinki, Helsinki, Finland; ^8^ Department of Plant Physiology, Umeå Plant Science Centre, Umeå University, Umeå, Sweden; ^9^ Department of Forest Genetics and Plant Physiology, Umeå Plant Science Centre, Swedish University of Agricultural Sciences, Umeå, Sweden

**Keywords:** Norway spruce, respiratory burst oxidase homolog (RBOH), lignin formation, hydrogen peroxide, calcium ion, phosphorylation

## Abstract

Plant respiratory burst oxidase homologs (RBOHs) are plasma membrane-localized NADPH oxidases that generate superoxide anion radicals, which then dismutate to H_2_O_2_, into the apoplast using cytoplasmic NADPH as an electron donor. *PaRBOH1* is the most highly expressed *RBOH* gene in developing xylem as well as in a lignin-forming cell culture of Norway spruce (*Picea abies* L. Karst.). Since no previous information about regulation of gymnosperm RBOHs exist, our aim was to resolve how PaRBOH1 is regulated with a focus on phosphorylation. The N-terminal part of PaRBOH1 was found to contain several putative phosphorylation sites and a four-times repeated motif with similarities to the Botrytis-induced kinase 1 target site in Arabidopsis AtRBOHD. Phosphorylation was indicated for six of the sites in *in vitro* kinase assays using 15 amino-acid-long peptides for each of the predicted phosphotarget site in the presence of protein extracts of developing xylem. Serine and threonine residues showing positive response in the peptide assays were individually mutated to alanine (kinase-inactive) or to aspartate (phosphomimic), and the wild type *PaRBOH1* and the mutated constructs transfected to human kidney embryogenic (HEK293T) cells with a low endogenous level of extracellular ROS production. ROS-producing assays with HEK cells showed that Ca^2+^ and phosphorylation synergistically activate the enzyme and identified several serine and threonine residues that are likely to be phosphorylated including a novel phosphorylation site not characterized in other plant species. These were further investigated with a phosphoproteomic study. Results of Norway spruce, the first gymnosperm species studied in relation to RBOH regulation, show that regulation of RBOH activity is conserved among seed plants.

## Introduction

Conifers are woody land plants that dominate vast areas in terrestrial ecosystems especially in the Northern Hemisphere. They contribute to a large fraction of biomass and act as an important carbon sink, the carbon being mainly allocated to cell walls of wood tissues. Norway spruce (*Picea abies* L. Karst.) is one of the most economically important coniferous species with primary significance for the wood-processing industry and as a carbon storage. It is also a model gymnosperm species whose whole genome sequence has been revealed (Nystedt et al., 2013). In its secondary xylem (wood), cell walls contain ca. 27% of a phenolic polymer, lignin (Jaakkola et al., 2007). Lignin provides rigidity and structural support to cell wall polysaccharides.

The final polymerization steps in lignin biosynthesis take place *via* H_2_O_2_-utilizing peroxidase- or O_2_-utilizing laccase-catalyzed oxidation of monolignols to phenolic radicals that then couple non-enzymatically as shown in Arabidopsis (*Arabidopsis thaliana*; Berthet et al., 2011; Novo-Uzal et al., 2013; Zhao et al., 2013; Shigeto and Tsutsumi, 2016; Dixon and Barros, 2019; Hoffmann et al., 2020; Rojas-Murcia et al., 2020; Blaschek and Pesquet, 2021; Blaschek et al., 2022), and various *Populus* species (Li et al., 2003; Lu et al., 2013; Lin et al., 2016; Qin et al., 2020). The relative contribution of these enzymes in lignification in the vasculature of plants *in vivo* and the specific isoenzymes that oxidize monolignols are not clearly known (Tobimatsu and Schuetz, 2019; Hoffmann et al., 2020; Hoffmann et al., 2022). In Arabidopsis, laccase and peroxidase isoenzymes localize differently in cell wall domains, with certain isoenzymes in the secondary cell walls of fibers and vessel elements, and the other ones in cell corners and middle lamella (Chou et al., 2018; Hoffmann et al., 2020). Recently, quadruple and quintuple loss of function laccase mutants of Arabidopsis were used to show that combinations of laccase isoenzymes are responsible for lignification in specific cell types and in different cell wall layers of primary xylem (Blaschek et al., 2022).

Localized apoplastic H_2_O_2_ production, required for the peroxidase-mediated lignification, is likely the regulatory factor that restricts the activity of peroxidases to certain locations in the lignifying cell wall (Hoffmann et al., 2020; Hoffmann et al., 2022). In the Casparian strip of the root endodermis of Arabidopsis, peroxidases have the main role in lignin formation (Lee et al., 2013). Mutation of five endodermal peroxidases leads to a total absence of lignification in the Casparian strip, whereas simultaneous abolishment of nine endodermis-expressed laccases has no effect on lignin formation (Rojas-Murcia et al., 2020). Hence, it seems that distinct enzymes are responsible for monolignol oxidation in various physiological phenomena and during different developmental stages.

During xylem development in Norway spruce, co-expression studies with monolignol biosynthesis genes raise both peroxidases and laccases as candidates for monolignol oxidation (Jokipii-Lukkari et al., 2017; Jokipii-Lukkari et al., 2018; Blokhina et al., 2019). In a Norway spruce cell culture that makes extracellular lignin (e.g. Simola et al., 1992; Kärkönen et al., 2002; Kärkönen and Koutaniemi, 2010), peroxidases seem to have the major role in monolignol activation since H_2_O_2_ scavenging from the medium efficiently inhibits lignin polymerization (Laitinen et al., 2017). Peroxidases need a source of apoplastic H_2_O_2_ used as an oxidant in monolignol activation, pointing out the role of enzymes able to generate apoplastic H_2_O_2_ in lignin polymerization.

Several plant cell wall- and plasma membrane-located sources can generate reactive oxygen species (ROS) such as superoxide anion radical, H_2_O_2_ and hydroxyl radical into the apoplast (Kärkönen and Kuchitsu, 2015; Smirnoff and Arnaud, 2019). These include respiratory burst oxidase homologues (RBOHs, also called NADPH oxidases) as well as various oxidases and peroxidases. RBOHs contain *heme* and flavin to utilize cytosolic NADPH as an electron donor and reduce O_2_ in the apoplastic side of the plasma membrane to superoxide (Torres and Dangl, 2005; Suzuki et al., 2011). As an enzyme to produce potentially toxic substances, both the enzymatic activity and expression of RBOHs are strictly regulated (Kurusu et al., 2015; Jiménez-Quesada et al., 2016). Superoxide dismutates to H_2_O_2_ and O_2_ in a reaction catalyzed by superoxide dismutase (Ogawa et al., 1997; Karlsson et al., 2005) or non**-**enzymatically in the acidic pH of cell walls. Arabidopsis has ten *RBOH* genes (*AtRBOH*s), and the different isoenzymes perform various functions and participate in distinct signaling pathways (Miller et al., 2009; Suzuki et al., 2011; Waszczak et al., 2018; Kaya et al., 2019; Castro et al., 2021). Intensive studies in a model species Arabidopsis have shown various regulatory mechanisms of RBOHs, that are mentioned in the following paragraphs.

Proteomic studies have revealed *in vivo* phosphorylation of AtRBOHD in pathogen-induced defense and abiotic stress responses (Nühse et al., 2007; Kadota et al., 2014; Wang et al., 2020). Various types of protein kinases such as SNF1-related kinase 2 protein kinases such as open stomata 1 kinase (Ost1; Sirichandra et al., 2009), Ca^2+^-dependent protein kinases (CPKs; Dubiella et al., 2013), Ca^2+^-activated calcineurin B-like interacting protein kinases (CIPKs; Kimura et al., 2013; Drerup et al., 2013; Han et al., 2019), receptor-like cytoplasmic kinases such as Botrytis-induced kinase 1 (BIK1; Li et al., 2014; Kadota et al., 2014; Kadota et al., 2015) and RPM1-induced protein kinases (RIPK, Li et al., 2021), mitogen-activated protein kinase kinase kinase kinases (MAP4K) such as SIK1 (Zhang et al., 2018), L-type lectin receptor-like kinases such as DORN1 (Chen et al., 2017), and a cysteine-rich receptor-like protein kinase 2 (CRK2, Kimura et al., 2020) have been shown to contribute to activation of RBOHs by phosphorylating distinct amino acids. Fine-tuning ROS production in various physiological situations is facilitated by protein kinase-specific target sites in addition to some common target sites (Kadota et al., 2015; Chen et al., 2017; Han et al., 2019). Since various protein kinases are activated by different factors (e.g., stress-induced Ca^2+^-signaling, pathogen- or damage-associated molecular patterns, i.e., PAMPs/DAMPs), target sites specific for certain kinases allow fine-tuning of ROS production by the same RBOH enzyme. Recently, a specific phosphatase contributing to the regulation of the RBOH activity was characterized (Han et al., 2019).

RBOHs have conserved Ca^2+^-binding EF-hand motifs at the N-terminus (Oda et al., 2010). Elevating the cytosolic Ca^2+^ concentration induced by pathogens or damage is essential for RBOH-mediated ROS production i*n planta* (Ranf et al., 2011; Kadota et al., 2014). Heterologous expression of RBOHs in HEK293T cells confirmed activation by Ca^2+^-binding to the EF-hand motifs (Ogasawara et al., 2008). Furthermore, Ca^2+^ binding and phosphorylation synergistically enhance the activity of RBOHs (Ogasawara et al., 2008; Takeda et al., 2008; Kimura et al., 2012; Han et al., 2019; Kaya et al., 2019). In addition to Arabidopsis, RBOH activity is regulated by Ca^2+^ and phosphorylation in monocotyledonous species such as rice (*Oryza sativa*; Takahashi et al., 2012).

Recent studies have shown that nitrosylation and persulfidation of cysteine residues at the C-terminus of AtRBOHD regulate the activity negatively and positively, respectively (Yun et al., 2011; Shen et al., 2020). Stability of AtRBOHD protein is controlled by ubiquitination at the C-terminus (Lee et al., 2020). Interaction with other regulatory proteins have also been shown (Kawarazaki et al., 2013; Kadota et al., 2015). Several studies suggest an important role of nitric oxide (NO) in regulating ROS production and scavenging (Gupta et al., 2020).

Involvement of RBOHs in the production of H_2_O_2_ necessary for the peroxidase-mediated lignin formation has been suggested in some herbaceous plants. During vascular xylem lignification and in *in vitro* tracheary element formation in zinnia (*Zinnia elegans*), RBOHs have been suggested to contribute to ROS production (Ros Barceló, 1998; Karlsson et al., 2005; Pesquet et al., 2013). In Arabidopsis, ROS produced by AtRBOHD/AtRBOHF are involved in cell wall damage-induced ectopic lignin formation (Denness et al., 2011) as well as lignin brace formation of the abscission layer in floral organs (Lee et al., 2018). In the Casparian strip of the root endodermis, AtRBOHF is the responsible enzyme involved in ROS production needed for lignification (Lee et al., 2013; Barbosa et al., 2019; Fujita et al., 2020). However, for peroxidase-mediated lignin formation in the vascular tissues of woody plants, no specific enzymes responsible for H_2_O_2_ production have so far been characterized.

Both the lignin-forming cultured cells and developing xylem of Norway spruce contain several plasma-membrane-located enzymes with the ability to generate superoxide anion radicals (and hence H_2_O_2_) (Kärkönen et al., 2014). In the cell culture, micromolar levels of H_2_O_2_ exist in the culture medium during lignin formation (Kärkönen and Fry, 2006; Kärkönen et al., 2009). Pharmacological experiments suggest that at least two enzymatic systems are involved in H_2_O_2_ generation: the one containing a *heme*, such as a peroxidase, and the other one a flavin, like in RBOH (Kärkönen et al., 2009). These observations raised an interest to investigate how RBOH activity is regulated in Norway spruce.

**Figure 1:**
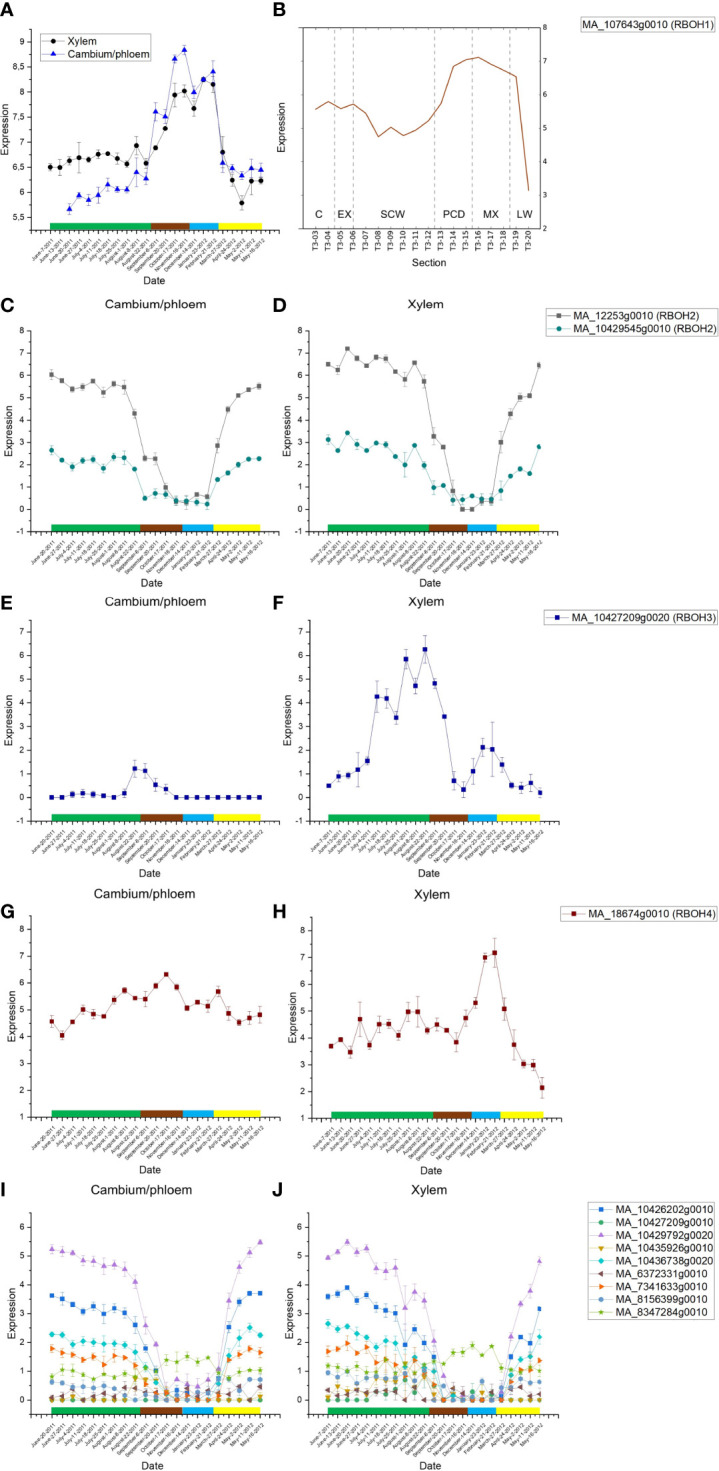
**(A)** Seasonal variation in the *PaRBOH1* expression in xylem and in combined cambium and phloem samples (data from Jokipii-Lukkari et al., 2018). *PaRBOH1* shows expression peak in late autumn and winter, and in mature xylem. Green rectangle, summer months June, July, and August; brown rectangle, autumn months September, October, and November; blue rectangle, winter months December, January, and February; and yellow rectangle, spring months March, April, and May. Values are means ± S.E. **(B)** Spatial expression pattern of *PaRBOH1* in the tangential section series across cambial region and current year wood. The data are retrieved from the NorWood web resource (http://NorWood.ConGenIE.org; Jokipii-Lukkari et al., 2017). The values correspond to variance stabilized gene expression. C, cambium; EX, xylem expansion; SCW, secondary cell wall formation; PCD, programmed cell death; MX, mature xylem; LW, previous year’s latewood. **(C–J)** Seasonal gene expression patterns of other Norway spruce *PaRBOH* genes in combined cambium and phloem samples **(C, E, G, I)** and in xylem **(D, F, H, J);** data from Jokipii-Lukkari et al. (2018).

Thirteen putative *RBOH* genes (*PaRBOHs*) are expressed in developing xylem of Norway spruce (Jokipii-Lukkari et al., 2017; Jokipii-Lukkari et al., 2018; http://congenie.org/; **Figure 1**). Among them, *PaRBOH1* is the most highly expressed *RBOH* gene in the tissue-cultured Norway spruce cells during extracellular lignin formation (Laitinen et al., 2017). We retrieved data from Jokipii-Lukkari et al. (2017); Jokipii-Lukkari et al. (2018) to demonstrate that this gene has high expression also in developing xylem of mature trees (**Figure 1A**). Furthermore, it has high expression in developing xylem cells during cell wall lignification (**Figure 1B**).

In the pre*s*ent study, we focused on PaRBOH1 to characterize its ROS-producing activity and regulatory mechanisms for the first time in gymnosperms including coniferous species. We showed that the developing xylem of Norway spruce has a protein kinase activity to phosphorylate some specific sites of PaRBOH1. PaRBOH1 was shown to be activated by the phosphorylation and binding of Ca^2+^ to the EF-hand motifs synergistically. Comparison with angiosperm RBOHs as well as possible significance in lignification in xylem development will be discussed.

## Material and methods

### Cloning full-length cDNA of *PaRBOH1*

Degenerate primers were designed based on an alignment of several plant *RBOH* genes available in the NCBI database. Total RNA was isolated according to Chang et al. (1993) from the tissue-cultured spruce cells and from the developing xylem of a mature tree of Norway spruce (*Picea abies* L. Karst.). RNA was reverse transcribed to cDNA with SuperScript III reverse transcriptase according to manufacturer’s instructions (Life Technologies Europe BV). Partial *RBOH* cDNA sequences were amplified with a polymerase chain reaction using the degenerate primers and Advantage 2 polymerase mix (Takara Bio). Rapid amplification of cDNA ends (3’ and 5’RACE) was done using SMARTer RACE 5’/3’ Kit (Takara Bio). PCR products were ligated to a pGEM-T easy vector (Promega) or to a pCR Blunt vector (Thermo Fisher Scientific) and transformed to DH5α competent *Escherichia coli* cells. With the full-length construct, it was necessary to cultivate the transformed cells at 28°C over two nights, since at 37°C the open reading frame was always disrupted with point mutations. The *PaRBOH1* coding sequence was submitted to a NCBI GenBank with an accession number HQ592777.2. Primers used for cloning are shown in **Supplementary Table S1**.

### Online software predictions for possible post-translational modifications in the PaRBOH1 N-terminus

In order to identify possible post-translational modification (PTM) sites and other motifs in the N-terminal 400 amino acid residues’ stretch of PaRBOH1 that exist before the transmembrane domains, several online databases and software tools for predicting protein PTMs were used -ScanProsite (de Castro et al., 2006; http://prosite.expasy.org/scanprosite); MotifScan (Pagni et al., 2007; http://myhits.isb-sib.ch/cgi-bin/motif_scan); P3DB (Yao et al., 2012a; Yao et al., 2014; http://www.p3db.org) together with Musite (Gao et al., 2010; Yao et al., 2012b; http://musite.net); PlantPhos (Lee et al., 2011; http://csb.cse.yzu.edu.tw/PlantPhos); DeepNitro (Xie et al., 2018; http://deepnitro.renlab.org/webserver.html). Where available, plant reference databases were used for the predictions. The outcomes from different tools were then compared, combined with the experimental evidence for PTMs of RBOH family members from other plant species.

### Cloning, expression and purification of the N-terminal part of PaRBOH1

In order to be used as a kinase substrate in *in vitro* kinase reactions, the 400 amino acid N-terminal part of PaRBOH1 was recombinantly expressed in *E. coli*. A T7*lac*-controlled expression system was used. Briefly, the N-terminal region was amplified by PCR with forward 5’–AGTAGCATATGGGTTTTGGTAC–3’ (incorporated NdeI site underlined) and reverse 5’–TTTCTCGAGGTTGTCTTCCACA–3’ (incorporated XhoI site underlined) primers, using cDNA of *PaRBOH1* as a template. The PCR product was purified by precipitation with 0.3 M (final concentration) sodium acetate and washing the pellet with EtOH, after which it was dissolved in water. The PCR product was digested with NdeI and XhoI, and ligated into a similarly-digested pET32a(+) plasmid (Novagen). The insertion removed a Trx-tag, an N-terminal 6xHis-tag and a S-tag as well as proteases’ cleavage sites and a multiple cloning site, and placed the open reading frame of the N-terminal sequence in frame with the codons of the C-terminal 6xHis-tag of the vector. After confirming the construct by sequencing, it was transformed into Rosetta™ 2 (DE3) competent cells (Novagen), chosen as a suitable bacterial expression system due to multiple rare codons in the coding sequence of the spruce gene. Large-scale expression of the PaRBOH1 N-terminus was performed at 25°C using the T7 auto-induction medium according to Studier (2005). The produced N-terminus was extracted from the bacterial cells with 8 M urea, captured on Ni-NTA agarose beads (QIAGEN), purified and eluted following QIAGEN’s protocol for 6xHis-tagged protein batch purification under denaturing conditions. The protein was buffer-exchanged in PD-10 gel-filtration columns (GE Healthcare) into 25 mM MOPS-KOH, pH 7.2.

### *In vitro* kinase assays: Preparation of crude protein extracts from developing xylem

Developing xylem of Norway spruce was collected from ca. 40 y-old trees grown in Ruotsinkylä, southern Finland in mid-/late June during active secondary growth as described in Väisänen et al. (2018), snap-frozen in liquid nitrogen, transported to a laboratory in dry ice, and stored at -80°C. The frozen samples were ground to a fine powder using an analytical mill (IKA A 11 basic, cooled with liquid nitrogen, 3 x 20 sec bursts), and stored at -80°C until use. To extract cytoplasmic proteins, the frozen xylem powder was added step-wise into a homogenization buffer (50 mM MOPS-KOH, pH 7.5, supplemented with 60 mM NaCl, 1 mM CaCl_2_, 0.1% (w/v) Triton X-100, 1.5% (w/v) polyvinylpolypyrrolidone (Polyclar AT), freshly added 5 mM dithiothreitol (DTT) and protease (Complete Mini EDTA-Free, Roche) and phosphatase (PhosSTOP, Roche) inhibitor cocktail tablets; 3 ml buffer/1 g cells). The mixture was incubated for 30 min on ice with occasional mixing, after which the large particles were pelleted by centrifugation (17 000 g, 10 min at 4°C). The supernatant was used immediately, or aliquoted and snap-frozen in liquid nitrogen and stored at -80°C until further assays.

### *In vitro* kinase assays with PaRBOH1 N-terminus and short synthetic peptides

*In vitro* kinase assays were conducted similarly for the 400 amino acid long N-terminus of PaRBOH1 and for the 15 amino acid peptides with each predicted phosphorylation site (see below). In order to resolve the amino acids being phosphorylation target sites, short synthetic peptides were designed based on the summary of *in silico* predicted phosphorylation sites. Peptides of 15 (in one case 14) amino acid residues with N-terminal biotinylation and C-terminal amidation were custom-produced (ProteoGenix, Schiltigheim, France; ca. 70% purity). To avoid possible steric hindrance with biotin and the spruce kinase(s), putative phosphorylation target sites were designed to always be the tenth (one time ninth) residue in the peptides (Curran et al., 2011). Lyophilized peptide aliquots were suspended in 50% acetonitrile (to 5 mM stocks), from which 0.1 mM working dilutions were prepared in 25 mM MOPS-KOH, pH 7.2. The kinase reactions (50 µl total volume) contained MOPS-KOH, pH 7.2 (30 mM final concentration), 2 µg recombinant N-terminus or 20 µM synthetic peptide (or 25 mM MOPS-KOH pH 7.2 in blank controls), 10 µl crude cytoplasmic protein extract from developing xylem as a kinase source, 1 mM DTT (added fresh), 25 mM β-glycerophosphate, additives (5 mM CaCl_2_, 5 mM MgCl_2_, 5 mM MnCl_2_, 2 mM EDTA and 5 mM EGTA in intended combinations) and 50 µM ATP (providing 10 kBq ^33^P[γ-ATP] per reaction when radioactivity was used in detection). The reaction was started by addition of ATP, and incubated at 25°C for 30 min. The reactions with the N-terminus were stopped by addition of the Laemmli buffer followed by SDS-PAGE electrophoresis and staining with ProQ Diamond phosphoprotein gel stain (Molecular Probes/Invitrogen) or autoradiography. *In vitro* kinase peptide assays were stopped by dilution with 250 µl of ice-cold 25 mM MOPS-KOH, pH 7.2, and 5 µl of streptavidin agarose ultra-performance beads (SoluLink, USA) were added. After incubation at 4°C for one hour with occasional mixing, the beads were pelleted by centrifugation for 10 min at 17 000 g, and the supernatant was removed. After two washes with 500 µl ice-cold 25 mM MOPS-KOH, the beads were resuspended in 300 µl 25 mM MOPS-KOH, pH 7.2, and the whole reactions were transferred into scintillation vials containing 2.7 ml Optiphase Hisafe 3 scintillation fluid (PerkinElmer). Radioactivity was detected on a Wallac 1414 WinSpectral liquid scintillation counter (PerkinElmer) using the ^45^Ca internal reference library. The signal in the blank controls was considered as a background, whose average value was subtracted from the sample values before calculations.

### HEK cell assays to study regulation of PaRBOH1 activity

To study whether PaRBOH1 produces superoxide *in cellula*, and how its activity is regulated, the protein was heterologously expressed in human embryonic kidney (HEK) cells (line HEK293T) according to Kaya et al. (2019) and Kimura et al. (2022). HEK293T cells have low endogenous ROS production since they lack NADPH oxidases 2 and 5 (Ogasawara et al., 2008). HEK cells were maintained at 37°C in 5% CO_2_ in Dulbecco’s Modified Eagle’s Medium nutrient mixture Ham’s F12 (Wako) supplemented with 10% fetal bovine serum (HyClone). A coding sequence of *PaRBOH1* was inserted in a pcDNA3.1-Kozak-3xFLAG vector and transfected by using GeneJuice transfection reagent (Novagen) into HEK293T cells cultivated on 96-well plates (100 ng construct/well) according to manufacturer’s instructions. Production of PaRBOH1 protein in HEK cells was verified by Western blotting using an antibody against the FLAG tag. ROS-producing activity assays were conducted as described in Ogasawara et al. (2008). Briefly, the culture medium was removed, and the cells were washed with Hanks’ Balanced Salt Solution without any Ca^2+^ or magnesium (HBSS (-/-) buffer, Gibgo) after which the ROS assay solution containing horseradish peroxidase (Wako, 4 or 60 µg/ml) and L-012 sodium salt (Wako, 250 µM) in HBSS (-/-) buffer was added. After a stabilization period of 10 min, CaCl_2_ (1 mM final concentration) and ionomycin, a Ca^2+^ ionophore (Calbiochem, 1 µM final), were simultaneously injected into the assay medium. ROS production was monitored by a luminol-based chemiluminescence technique using L-012 as a substrate for peroxidase as described in Kaya et al. (2019). Similarly, the effects of a protein serine/threonine phosphatase inhibitor calyculin A (Wako; 0.1 µM final) were tested. Additionally, diphenylene iodonium (DPI; 0.1, 0.5 and 5 µM), an inhibitor of flavin-containing enzymes, and a protein kinase inhibitor K252a (0.1 and 1 µM) were individually added to treatment wells prior to the assays to evaluate their effects on ROS production. To study the effect of Ca^2+^, various concentrations of Ca^2+^ (0, 0.25, 0.5 and 1 mM) were added to assay wells immediately prior to the start of the experiment, and ionomycin (no Ca^2+^ supplementation with ionomycin in this case) injected at 20 min. Since ionomycin, calyculin A and DPI were originally dissolved in DMSO, the effects of the corresponding volume of DMSO were tested to see that the effects were not due to DMSO.

In order to identify the amino acids being phosphorylated, candidate amino acids showing positive response in the short peptide activity assays were individually mutated to alanine (A; kinase-inactive) and aspartate (D; phosphomimic) (Dean et al., 1989). The mutants were generated by point-mutation primers using the SPRINP mutagenesis protocol (Edelheit et al., 2009). The primer sequences used in this study are shown in **Supplementary Table S2**. The full-length mutated constructs were tested in the HEK cell assays, and the ROS-producing activity compared to that of the non-mutated PaRBOH1 assayed in the same experiment. All experiments were conducted independently for at least three times with a similar trend in results.

### Phosphoproteomic analysis of PaRBOH1

To determine the phosphorylated amino acids in the native PaRBOH1 during lignin formation *in planta*, microsomal membrane fraction (MF) of developing xylem was isolated (Kärkönen et al., 2014; Väisänen et al., 2018), proteins solubilized with 4% SDS and separated in an SDS-PAGE gel (with loading in every well). Proteins were transferred to a nitrocellulose membrane, and a Western blot conducted to membrane slices that located in both edges of the gel by using a custom-made antibody against PaRBOH1 (GenScript). By knowing the location of the PaRBOH1, the corresponding bands were cut out from the membrane, and the protein sent for proteomic analysis (see below).

### *In vitro* kinase reactions using soluble and membrane-bound kinases from developing xylem

To determine phosphotarget sites for spruce endogenous kinases, an *in vitro* kinase reaction was conducted in a reaction that contained 25 mM MOPS-KOH, pH 7.2, 1 mM DTT, 25 mM β-glycerophosphate, 2 mM CaCl_2_, 15 mM MgCl_2_, 2 mM MnCl_2_, phosSTOP (Roche), a protease inhibitor (EDTA-free, Roche), 10 mM NaATP (fresh, pH adjusted to neutral), 91.7 µg PaRBOH1 N-terminus and 500 µl of freshly prepared xylem cell extract in a final reaction volume of 2.5 ml. The reaction mixture was gently mixed and incubated at 25°C in the dark for 2 h with occasional mixing. After the reaction, the N-terminus was buffer-exchanged in an PD-10 column to phosphate buffer (12.8 mM Na-phosphate, pH 7.4, supplemented with 6 M urea and 320 mM NaCl). The eluate (3.1 ml) was mixed with an equal volume of Ni-NTA resin containing imidazole (1.25 mM final concentration) and incubated overnight at 10°C with slow (50 rpm) shaking, pipetted into two Qiagen columns (3.1 ml/column) at room temperature, and the flow-through collected. The resin was first washed with 6.2 ml of 2.5 mM imidazole in phosphate buffer; then with 6.2 ml of 5 mM imidazole in phosphate buffer. The bound proteins were eluted with 500 mM imidazole in phosphate buffer. Comparable eluates from two columns were combined and concentrated with an Amicon Ultra-4 10K centrifugal filter device (2800 g, 4°C, 30 min). Subsequently, the concentrated N-terminus was re-purified with the Ni-NTA resin. The proteins were separated in an SDS-PAGE gel, and pieces of the gel corresponding to the location of the N-terminus cut out for proteomic analysis. As a control, the PaRBOH1 N-terminus that did not go through any kinase reaction was separated in an SDS-PAGE gel, cut out and analyzed.

For the *in vitro* membrane-bound kinase reactions, a fresh MF containing all cellular membranes was prepared from Norway spruce developing xylem as described in Kärkönen et al. (2014) and Väisänen et al. (2018) with additional 50 mM β-glycerophosphate in the homogenization buffer. The MF obtained was washed by resuspending the pellets in 10 mM MOPS-KOH, pH 7.5, and the membranes were collected by ultracentrifugation (Beckman, 50.2Ti rotor, 200 000 g, 35 min at 4°C). The pellets were resuspended to 10 mM MOPS-KOH, pH 7.5, in a final volume of ca. 1 ml. An *in vitro* kinase reaction was conducted as described above for the soluble kinases except that 500 µl of freshly prepared xylem MF was used as a source of kinases in a final reaction volume of 2.5 ml. After 2 h, the membranes were pelleted (17 000 *g*, 30 min at 4°C). Surprisingly, the PaRBOH1 N-terminus was observed not to be soluble in the supernatant but pelleted with the MF membranes. Addition of increasing concentration of NaCl (up to 3 M in 10 mM MOPS-KOH, pH 7.5) was not able to release the N-terminus from the membranes, and thus, the membranes were incubated 10 min in 4% SDS at room temperature, centrifugated 10 min (1700 g at room temperature), and the supernatant collected. SDS was shown to liberate the N-terminus from the membranes (data not shown). To purify the N-terminus for phosphoproteomic analysis, the SDS-liberated proteins were buffer-exchanged in MidiTrap columns to 12.8 mM Na-phosphate buffer, pH 7.4, supplemented with 6 M urea and 320 mM NaCl, purified in Ni-NTA resin, and separated in an SDS-PAGE gel like described above. There was a fainter band migrating slightly slower (band 2) than the N-terminus (band 1; **Supplementary Figure S1).** Since phosphorylation increases the molecular weight, both bands were cut out for proteomic analysis.

### Phosphoproteomic analysis

Excised protein gel band pieces were reduced and alkylated with 20 mM DTT and 50 mM iodoacetamide successively. The proteins were digested by adding 0.5 µg trypsin (Promega) in 50 mM ammonium hydrogen carbonate, and the mixture was incubated overnight at room temperature (Vaarala et al., 2014). The peptides were cleaned using C18-reverse phase ZipTip™ (Millipore), resuspended in 1% trifluoroacetic acid (TFA) and sonicated in a water bath for 1 min.

Phosphopeptides in the digest were either directly detected by LC-MS^E^ or were TiO_2_-enriched. Phosphopeptide enrichment was done using TiO_2_ cartridges (Agilent Technologies) following the manufacturer’s protocol. In brief, TiO_2_ cartridges were primed and equilibrated with 50% acetonitrile (ACN)/45% water/5% NH_3_ and 50% ACN/48% water/2% TFA successively. Peptides were bound, washed with equilibration solution and ultimately eluted with 80% H_2_O/15% ACN/5% NH_3_.

Digested proteins were injected for LC-MS^E^ analysis. The peptides were separated by Nano Acquity UPLC system (Waters) equipped with a Symmetry C18 reverse phase trapping column (180 μm × 20 mm, 5 μm particles; Waters), followed by an analytical BEH-130 C18 reversed-phase column (75 µm × 250 mm, 1.7 µm particles; Waters), in a single pump trapping mode. The injected sample analytes were trapped at a flow rate of 15 µl min^-1^ in 99.5% of solution A (0.1% formic acid, FA). After trapping, the peptides were separated with a linear gradient of 3 to 35% of solution B (0.1% FA/ACN), for 30 min at a flow rate 0.3 µl min^-1^ and a stable column temperature of 35°C. The samples were run in a data-independent analysis mode (MS^E^) in a Synapt G2-S mass spectrometer (Waters), by alternating between low collision energy (6 V) and high collision energy ramp in the transfer compartment (20 to 45 V) and using a 1-sec cycle time. The separated peptides were detected online with a mass spectrometer, operated in a positive resolution mode in the range of *m/z* 50-2000 amu. Human [Glu^1^]-fibrinopeptide B (Sigma, 150 fmol µl^-1^) in 50% ACN/0.1% FA solution at a flow rate of 0.3 µl min^-1^ was used for lock mass correction, applied every 30 sec (Scifo et al., 2015).

Waters’ ProteinLynx Global Server (PLGS V3.0) software was used to search for protein identifications. MS^E^ parameters were set as follows: low energy threshold of 135 counts, elevated energy threshold of 30 counts, and intensity threshold of precursor/fragment ion cluster 750 counts. Database searches were carried out against *Picea abies, NCBInr* (release of May 2019; 28233 entries) with Ion Accounting algorithm and using the following parameters: peptide and fragment tolerance: automatic; maximum protein mass: 1000 kDa; minimum fragment ions matches per protein ≥ 7; minimum fragment ions matches per peptide ≥ 3; minimum peptide matches per protein ≥ 2; primary digest reagent: trypsin; missed cleavages allowed: 1; fixed modification: carbamidomethylation (C); variable modifications: phosphorylation (STY), and false discovery rate (FDR) < 4%. The method workflow was tested and validated beforehand by using β-casein for in-gel digestion and LC-MS^E^ analysis. The raw data of HDME LCMS runs, the PLGS 3.0 analysis result files, and the protein database used in the searches in ProteomeXchange (identifier PXD035518) are provided via Massive Spectrometry Interactive Virtual Environment (MassIVE Dataset): http://massive.ucsd.edu/ProteoSAFe/status.jsp?task=a3bd84481aa74996be9278bf1084c858

### Construction of a PaRBOH1 hairpin for gene silencing

A ca. 300 bp DNA fragment (including the coding area for the repetitive motif) of the *PaRBOH1* cDNA (GenBank HQ592777.2) was amplified by PCR, using Phusion high-fidelity DNA polymerase (New England BioLabs) and forward 5’-TGTGGATAGAGTTGCCTTCAG-3’ and reverse 5’-TCCACATATCCTTCATCTCCA-3’ PCR primers, with attB sites for Gateway (Life Technologies) cloning added at their 5’ ends. The gel-purified PCR product was inserted *via* Gateway BP recombination into pDONR™221 (Life Technologies) to create a pENTRY-*PaRBOH1*-i plasmid. After confirmation by sequencing, the *PaRBOH1* fragments were transferred *via* Gateway LR recombination to a custom-made plasmid with two attR cassettes in the opposite orientation (a generous gift from Ove Nilsson, Umeå Plant Science Centre, UPSC, Sweden), resulting in a “*PaRBOH1*-i-hairpin” construct, i.e., two inverted PaRBOH1 insertions separated by the maize ubiquitin intron. This construct was verified by BplI digestion.

### Transformation of spruce embryogenic cultures

*PaRBOH1*-i-hairpin construct was used for stable transformation of an embryogenic 11:12:04 Norway spruce cell line. Embryogenic spruce cells were co-cultivated with *Agrobacterium tumefaciens* strain LBA4404 following established protocols at the spruce transformation facility at UPSC (Sofie Johansson and Ove Nilsson, *pers. comm;* von Arnold and Clapham, 2008). Following co-cultivation, the filter paper disks with pro-embryogenic masses were transferred onto fresh solid ½ LP medium (0.35% gelrite) supplemented with 300 mg l^-1^ cefotaxime for the recovery, followed by cultivation in the dark for 7-10 days.

Selection for transformants was conducted on solid ½ LP medium supplemented with 300 mg l^-1^ cefotaxime with initially 1 mM (for two weeks) and subsequently with 3 mM d-serine (for two weeks) in the dark at 20-21˚C. Transgenic callus with vigorous growth was tested by genomic PCR for the presence of *d-serine ammonia lyase* (*dsdA)* gene from *E. coli* (Erikson et al., 2005). For somatic embryo regeneration, transformed embryogenic cultures were cultivated first on solid pre-maturation (DKM) medium (Krogstrup, 1986) without plant growth regulators but supplemented with 3 mM d-serine. After cultivation in the dark for a week, the calli were transferred for maturation on DKM medium supplemented with 29 µM ABA, 3% sucrose and 3 mM d-serine for a total of 6 weeks. Fully developed embryos were dried for 3 weeks under high humidity in the dark, and subsequently germinated on solid medium containing minerals, vitamins, 3% sucrose and 0.5 g l^-1^ casein hydrolysate, pH 5.8.

For germination, plates were placed under constant red light at 20°C, and after 2-3 weeks, the plates were moved to constant white fluorescent light (Kvaalen and Appelgren, 1999) until roots and shoots were fully developed (Dobrowolska et al., 2017). Plantlets were acclimatized under 24 h light (150 μmol m^-2^ s^−1^) at 20°C, and cultivated in a greenhouse under constant light for two years (with a short-day- and decreasing-temperature-induced dormancy in winter).

### Real-time quantitative RT-PCR

In total 19 2-y-old plants were sampled for RT-qPCR: five independent transformant lines each having three biological replicates and four control plants, one being a full control and three half controls. Bark was peeled off from the stems, and the xylem was ground into powder with a mortar and pestle in liquid nitrogen. Total RNA was extracted from the powder according to Chang et al. (1993), and RNA was dissolved in 20 µl of nuclease-free water. Residual genomic DNA was digested with DNase I, and RNA was cleaned using Nucleospin RNA Clean-up Mini kit (Macherey-Nagel) according to the manufacturer’s protocol. First-strand cDNA was synthesized using 1 µg of total RNA with SuperScript III reverse transcriptase (Invitrogen) according to manufacturer’s instructions.

Amplification efficiencies of primers were determined using dilution series of cDNA prepared by combining equal amounts of first-strand cDNA from control plants. Primers were accepted if the efficiency was at least 1.8 with the standard error of less than 5%. *Glyceraldehyde 3-phosphate dehydrogenase*, *adenosine kinase* and *actin* genes were used as internal controls. PCR mixture was prepared in a 15 µl reaction volume using LightCycler 480 SYBR Green I Master mix (Roche), 0.5 µM of primers and 5 µl of diluted first-strand cDNA. The RT-qPCR was done in a LightCycler 480 instrument (Roche) with following program: 95°C for 10 min, 45 cycles of 95°C for 10 s, 57°C for 10 s, and 72°C for 10 s followed by a melting curve analysis. The data were analyzed using two different methods described in Lim et al. (2016) and Pfaffl (2001).

## Results

### Protein kinase activity of the developing xylem of Norway spruce to phosphorylate peptides from PaRBOH1

**Figure 2:**
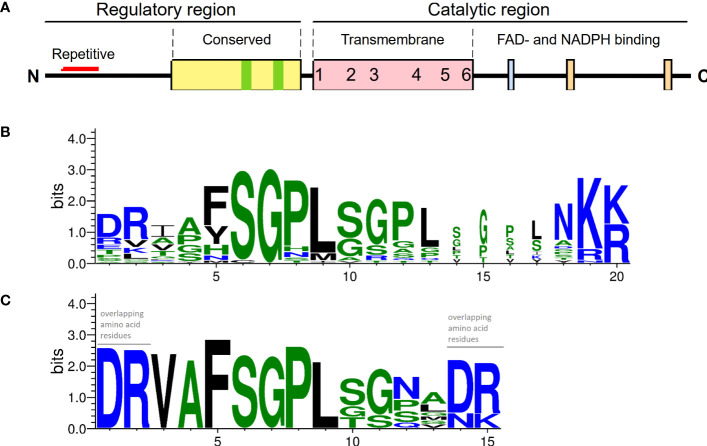
A primary structure of PaRBOH1. **(A)** A schematic model of the PaRBOH1 structure. **(B)** A sequence logo image of the conservation of the BIK1 target site corresponding to S39 in AtRBOHD in members of the plant *RBOH* gene family with up to three SGPL-like adjacent motifs (**Supplementary Table S3A**). **(C)** A sequence logo image of the BIK1 target site in the N-terminal repetitive motif generated using the four-times repeated motif from PaRBOH1 and the five-times repeated motif from its closest *P. taeda* homologue (**Supplementary Table S3B**). Sequence logos were generated by WebLogo (Crooks et al., 2004), hydrophilic charged (blue) and neutral (green) amino acid residues are indicated.

Sequence analysis showed that the N-terminal part of PaRBOH1 contains a four-times repeated motif whose sequence includes a SGPL-motif similar to that in the Botrytis-induced kinase 1 (BIK1) target site S39 in AtRBOHD (Kadota et al., 2014; **Figure 2**; **Supplementary Table S3**). In addition, the use of various *in silico* prediction programs resulted in identification of several candidate phosphorylation target sites in the N-terminus of PaRBOH1. A couple of putative nitrosylation sites were found in the transmembrane (C456) and in the C-terminal (C933) domains as well (**Supplementary Table S4**). *In vitro* protein kinase assays using the 400 amino acid N-terminus, non-labelled ATP or ^33^P[γ-ATP], and an extract of developing xylem as a source of soluble kinases indicated that phosphorylation(s) indeed occurred. To test which of the predicted sites were phosphorylated by spruce kinases, biotin-labelled, 15 amino acid long peptides were synthesized with each predicted phosphorylation target site in the 10^th^ (or 9^th^) position. *In vitro* kinase reactions were conducted with the peptides as kinase substrates and a crude extract of developing Norway spruce xylem as a kinase source. Out of 16 tested peptides, six showed a positive response in the *in vitro* kinase peptide assays (**Table 1**). In two of the positive peptides, two possible phosphorylation target sites located next to each other (T149 and S150; T174 and S175; **Table 1**), making the phosphosite assignment challenging.

**Table 1:** Phosphorylation of the PaRBOH1 N-terminal serine (S) and threonine (T) residues in *in vitro* kinase assays with short peptides and the full N-terminus, and the effect of phosphorylation of these residues on the enzymatic activity in HEK cell assays.

(A)			(B)	(C)		
Position in PaRBOH1	15-mer sequence	Response in *in vitro* kinase peptide assays	HEK cell assays	Phosphoproteomic analysis after *in vitro* kinase reactions		
				Soluble kinases	Membrane-bound kinases band 1	Membrane-bound kinases band 2
S32		n.d.	A: ±, D: ±	+	+	+
S45		n.d.	A: ±, D: ±	+^¶, #^	+^¶, #^	+^¶, #^
S58		n.d.	A: ±, D: ±	+^¶, #^	+^¶, #^	+^¶, #^
S71		n.d.	A: ±, D: ±	+^#^	+^#^	+^#^
S86	PLNKR PGRR **S** ARFNI	+	A: ±, D: ±			
T149	LAKDL EKK **T** S FGSSI	+^†^	A: ±, D: ±	+	+	+
S150	LAKDL EKKT **S** FGSSI	+^†^	A: +, D: ++	+	+	+
S160	FGSSI IRNA **S** ARIKH	+	A: -, D: ++			
T174	VSQEL KRL **T** S FTKRS	+^‡^	A: 0, D: +++	+		
S175	VSQEL KRLT **S** FTKRS	+^‡^	A: 0, D: 0		+	
T190	HPGRL DRSK **T** GAHHA	–	A: 0, D: ++			
T300§	VDKNA DGRI **T** EEEVK	+	A: -, D: +++			
S374	TSLNL SQMI **S** QKLVP	+	A: -, D: +++			

(A) Sequences of peptides with N-terminal biotinylation were designed with the predicted phosphorylation site at the position 9 or 10 in the 15-mer (underlined bold character). The peptides showing enhanced phosphorylation (+) as compared to no-peptide controls in at least two independent *in vitro* kinase experiments are shown; -, no difference as compared to no-peptide control; ^†,‡^ the phosphotarget site cannot be distinguished based on *in vitro* kinase peptide assays; n.d., not determined. (B) For HEK cell assays, each putative phosphotarget site was mutated to alanine (A)/aspartate (D), and the effects on apoplastic ROS production evaluated according to Kaya et al. (2019). 0, similar ROS production as in the wild-type construct; -, decreased ROS production as compared to the wild-type construct; ±, no consistent response as compared to the wild-type construct; +, ++ or +++, enhanced ROS production as compared to the wild-type construct. ^§^ putative phosphosite overlaps the first EF hand. (C) Phosphorylated residues detected in the phosphoproteomic analysis of the PaRBOH1 N-terminus after *in vitro* kinase reactions using soluble cytoplasmic extract or microsomal membrane fraction of developing xylem of Norway spruce as sources for kinases. Band 1 and band 2 (**Supplementary Figure S1**): two bands cut out from the SDS-PAGE gel after membrane-bound kinases were used in *in vitro* kinase reaction followed by SDS-PAGE to separate the PaRBOH1 N-terminus. See **Table 2** for details. ^¶^S45 and S58 cannot be distinguished in the phosphoproteomic analysis. ^#^ Some phosphorylation also in the control PaRBOH1 N-terminus that did not go through the *in vitro* kinase assay.

### ROS-producing activity of PaRBOH1 *in cellula*

**Figure 3:**
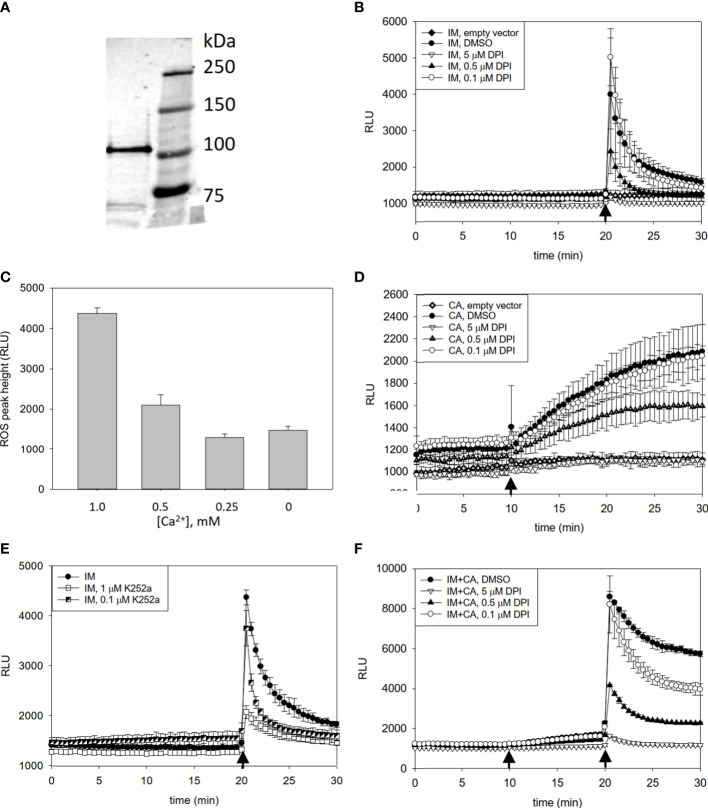
Detection of heterologously produced PaRBOH1 in HEK cells and effects of pharmacological inhibitors and Ca^2+^ on ROS production. **(A)** A protein of a correct size (107 kDa) is produced in HEK cells transfected with a *PaRBOH1* construct as revealed by a Western blot using an antibody against the FLAG-tag. **(B–F)** Effects of pharmacological inhibitors and Ca^2+^ on ROS production in human embryogenic kidney (HEK293T) cells transfected with *PaRBOH1.* **(B)** After measuring the baseline ROS production for 20 min, a Ca^2+^ ionophore ionomycin (IM; 1 µM) and 1 mM Ca^2+^ were simultaneously injected into the assay mixture. **(C)** Various concentrations of Ca^2+^ were externally added to assay wells just prior to the start of the experiment. IM was injected at 20 min. Maximum ROS peaks are shown. **(D)** After measuring the baseline ROS production for 10 min, a phosphatase inhibitor calyculin A (CA; 0.1 µM) was injected into the assay mixture. **(E)** Effect of a kinase inhibitor K252a on ROS production. K252a (0.1 or 1 µM) was supplemented to the assay mixture just prior to the start of an experiment. IM was injected at 20 min. **(F)** A combined effect of calyculin A and IM on ROS production. Calyculin A (0.1 µM) was injected at 10 min, and IM (1 µM) supplemented with 1 mM Ca^2+^ at 20 min. In Figure 3 **(B, D, F)**, an inhibitor of flavin-containing enzymes, diphenylene iodonium (DPI, 0.1, 0.5 or 5 µM), was supplemented to the assay mixture just prior to the start of an experiment. Control wells obtained a corresponding volume of DMSO. Data are expressed as relative luminescence units (RLU). Each data point represents the average of three wells analyzed in parallel (mean ± S.D.). The experiment was repeated for at least three times in separate experiments with similar trend in results. Arrows, time of injection.

To evaluate the regulation of PaRBOH1 activity *in cellula*, the wild type *PaRBOH1* gene was transiently expressed in HEK293T cells having a low level of endogenous extracellular ROS production. Even though the codon usage in conifers is partially different to that in humans, a protein of a correct size was shown to be produced in HEK cells (**Figure 3A**). Simultaneous injection of ionomycin, a Ca^2+^ ionophore (1 µM), and CaCl_2_ (1 mM) induced a transient peak of ROS production in the PaRBOH1-transfected cells, but not in the empty-vector-transfected control cells (**Figure 3B**). Moreover, addition of diphenylene iodonium (DPI; 0.1-5 µM), an inhibitor of flavin-containing enzymes, inhibited ROS production (**Figure 3B, D, F**), indicating the ROS-producing activity of PaRBOH1. Decreasing Ca^2+^ concentration diminished the height of the ROS peak (**Figure 3C**), suggesting that Ca^2+^ influx and the following binding of Ca^2+^ to the EF-hand motifs of PaRBOH1 triggered ROS production. Hence, 1 mM CaCl_2_ was used in the subsequent experiments.

Calyculin A, an inhibitor of protein phosphatases, increased the extracellular ROS levels from those in the DMSO-only treated cells (**Figure 3D**); this increase was stable for the 20-min experimental time after calyculin A addition. Vice versa, K252a, an inhibitor of a protein kinase, decreased the ionomycin-induced ROS peak in a concentration-dependent manner (**Figure 3E**). These observations suggest that protein phosphorylation is crucial in the regulation of the PaRBOH1 activity. Addition of calyculin A followed by an ionomycin-CaCl_2_ treatment led to the strongest ROS burst, indicating that similar to several other RBOHs (Ogasawara et al., 2008; Kimura et al., 2012; Kaya et al., 2019), phosphorylation enhances the induction of PaRBOH1 activity by Ca^2+^ (**Figure 3F**).

### Phosphorylation target sites in the PaRBOH1 N-terminus

**Figure 4:**
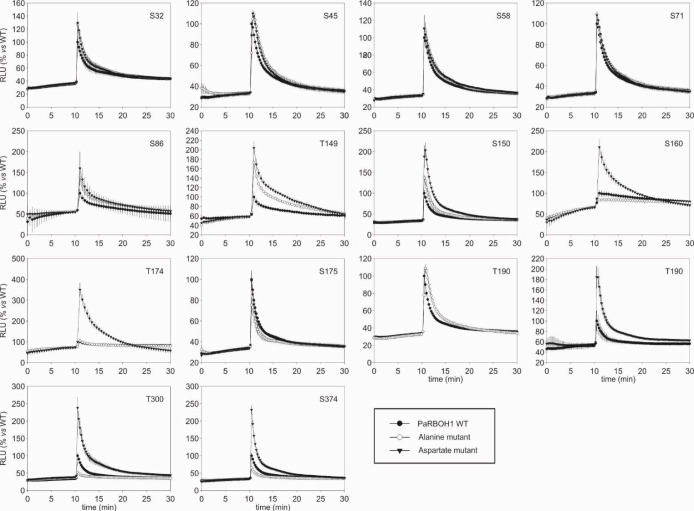
Impact of alanine (A) and aspartate (D) phosphosite substitution mutants of PaRBOH1 on ionomycin-induced ROS production in transfected HEK293T cells. ROS production was detected by luminol-amplified chemiluminescence. HEK cells were transiently transfected with *PaRBOH1* WT (wild type), *PaRBOH1* with serine/threonine-alanine mutation (A, kinase-inactive) or *PaRBOH1* with serine/threonine-aspartate mutation (D, phosphomimic) at the indicated PaRBOH1 phosphotarget site. ROS production activity of PaRBOH1 and its A and D mutants was measured in response to adding 1 µM ionomycin and 1 mM Ca^2+^ at 10 min and expressed as relative luminescence units (RLU; PaRBOH1 WT response was set to 100%). Results are means of three wells ± S.E. (n = 3). Similar results were obtained at least in three independent experiments.

To determine the phosphorylation target sites in the N-terminus responsible for regulation of the PaRBOH1 activity, the serine/threonine residues showing positive response in the *in vitro* kinase peptide assays and those in the repetitive sequence motif (**Table 1**) were individually mutated to alanine (A; kinase-inactive) and aspartate (D; phosphomimic), and the effects on ROS production were tested in HEK cells. Mutations of serine residues in the repetitive sequence motif (S32, S45, S58, S71; **Figure 2C**; **Supplementary Table S3B)** did not show any consistent differences in ROS production as compared to non-mutated, wild-type PaRBOH1 (**Table 1**; **Figure 4**). However, phosphoproteomic analysis of the PaRBOH1 N-terminus after *in vitro* kinase assays using spruce endogenous kinases from the developing xylem, on the other hand, showed that these serine residues, S32, S45/58 and S71, were phosphorylated by both soluble and membrane-bound protein kinases (**Tables 1** and **2**; **Supplementary Data S1**; **Supplementary Figures S2-S8**). In addition, several other serine and threonine residues were shown to be phosphorylated, with T149, S150 and T174 residues with the highest intensity (**Table 2**).

**Table 2:** Phosphoproteomic analysis of the N-terminus of PaRBOH1.

	Control		Cytoplasmic soluble kinases		Membrane-bound kinases			
					band 1		band 2	
**Residue**		**TiO_2_**		**TiO_2_**		**TiO_2_**		**TiO_2_**
**T5**		2735						
**T16**	5450		10915		944			
	**7.00%**		**22.60%**		**5.67%**			
**T22**		1270						
**S32**			18191	21437	40310	52839	23208	11650
			**0.18%**		**0.65%**		**1.73%**	
**S36**			10915	13627	9217	4516		
			**0.10%**		**0.15%**			
**S37**						11362		
**S45/S58**	1097		32518	58464	107010	180890	43497	31290
	**0.00%**		**0.14%**		**0.84%**		**1.52%**	
**T49**				2870				
**S71**	2169		25280	5296	41740	26641	11852	
	**0.02%**		**0.28%**		**1.00%**		**1.43%**	
**T75**					5959			
					**0.14%**			
**S119**	1641							
	**12.40%**							
**T149**			3703	175051	167858	306829	4416	34697
			**0.09%**		**29.78%**		**1.61%**	
**S150**			113920		14393		38805	
			**2.76%**		**1.04%**		**30.91%**	
**S154**						822		
**T174**			217769	452372				
			**23.90%**					
**S175**						52547		
**T177**				89328		39460		
**S188**							19713	
							**90.30%**	
**Y342**	1245							
	**3.24%**							
**S366**					4167			
					**1.30%**			

Phosphorylated residues detected in the phosphoproteomic analysis of the PaRBOH1 N-terminus after *in vitro* kinase reactions using soluble cytoplasmic extract or microsomal membrane fraction of developing xylem of Norway spruce as sources for kinases. Control: in *Escherichia coli* produced PaRBOH1 N-terminus without any *in vitro* kinase reaction; Band 1 and band 2: two bands cut out from the SDS-PAGE gel after membrane-bound kinases were used in *in vitro* kinase reaction followed by SDS-PAGE to separate the PaRBOH1 N-terminus (**Supplementary Figure S1**). In the table, the upper numbers represent the intensity in the LC-MS^E^ analysis, and the numbers below (in bold) represent the percentage of phosphorylated *versus* non-phosphorylated peptide. TiO_2_: titanium oxide enrichment.

Mutations of the putative target sites (serine or threonine residues) did not affect the protein amounts of PaRBOH1 (**Supplementary Figure S9**). In the HEK cell assays, however, mutation of the following putative target sites (serine or threonine residues) to either alanine or aspartate residues showed clear responses in ROS production (either decreased/unaffected in case of alanine substitution or enhanced in case of aspartate substitution): S150, S160, T174, T190, T300, S374 (**Figure 4**). Mutations of S86 or S175 did not affect the ROS-producing response in HEK cells, whereas that of T149 did not give consistent responses. Phoshoproteomic results after *in vitro* kinase assays using spruce kinases were consistent with few detected phosphosites in the HEK cell assays (S150, T174; **Tables 1** and **2**; **Supplementary Data S1**). S150 corresponds to the T123 site in AtRBOHD, which is also a target site for BIK1. To our knowledge, T174 is a novel regulatory site not yet described in other species. Interestingly, T174D construct was able to induce the highest ROS production (ca. 3.5 times higher than in the wild type PaRBOH1; **Figure 4**).

### Generation of *PaRBOH1*-silenced plants

To verify the function of PaRBOH1 *in planta*, a hairpin construct of *PaRBOH1* driven by a maize ubiquitin promoter was transformed to Norway spruce embryogenic cultures with the aim to silence the gene. After antibiotic selection, embryos were induced and plantlets regenerated from several putatively silenced lines originating from independent transformation events. Unfortunately, all but one of the non-transformed, genetically identical control plantlets (full-control) were lost. Hence, non-transformed plantlets from a genetically half-identical line were used as controls (half-controls). Due to differences in *PaRBOH1* expression in the full-control and half-control plants, the effects of gene silencing were difficult to conclude (data not shown). Thus, the role of PaRBOH1 in wood development still remains to be evaluated.

## Discussion

Oxidation of monolignols, one of the final steps of lignification, involves either peroxidases or laccases in angiosperm species. In spite of the critical importance of lignin biosynthesis in coniferous tree species, however, its molecular mechanisms remain partially unknown. Our earlier studies suggested that peroxidases and the ROS-producing RBOHs play a major role in lignification (Kärkönen et al., 2009; Kärkönen et al., 2014; Laitinen et al., 2017). We therefore characterized the ROS-producing activity and its regulatory mechanisms of the most highly expressed RBOH, PaRBOH1, in both the developing xylem (**Figure 1**) and the lignin-forming cell culture (Laitinen et al., 2017).

**Figure 5:**
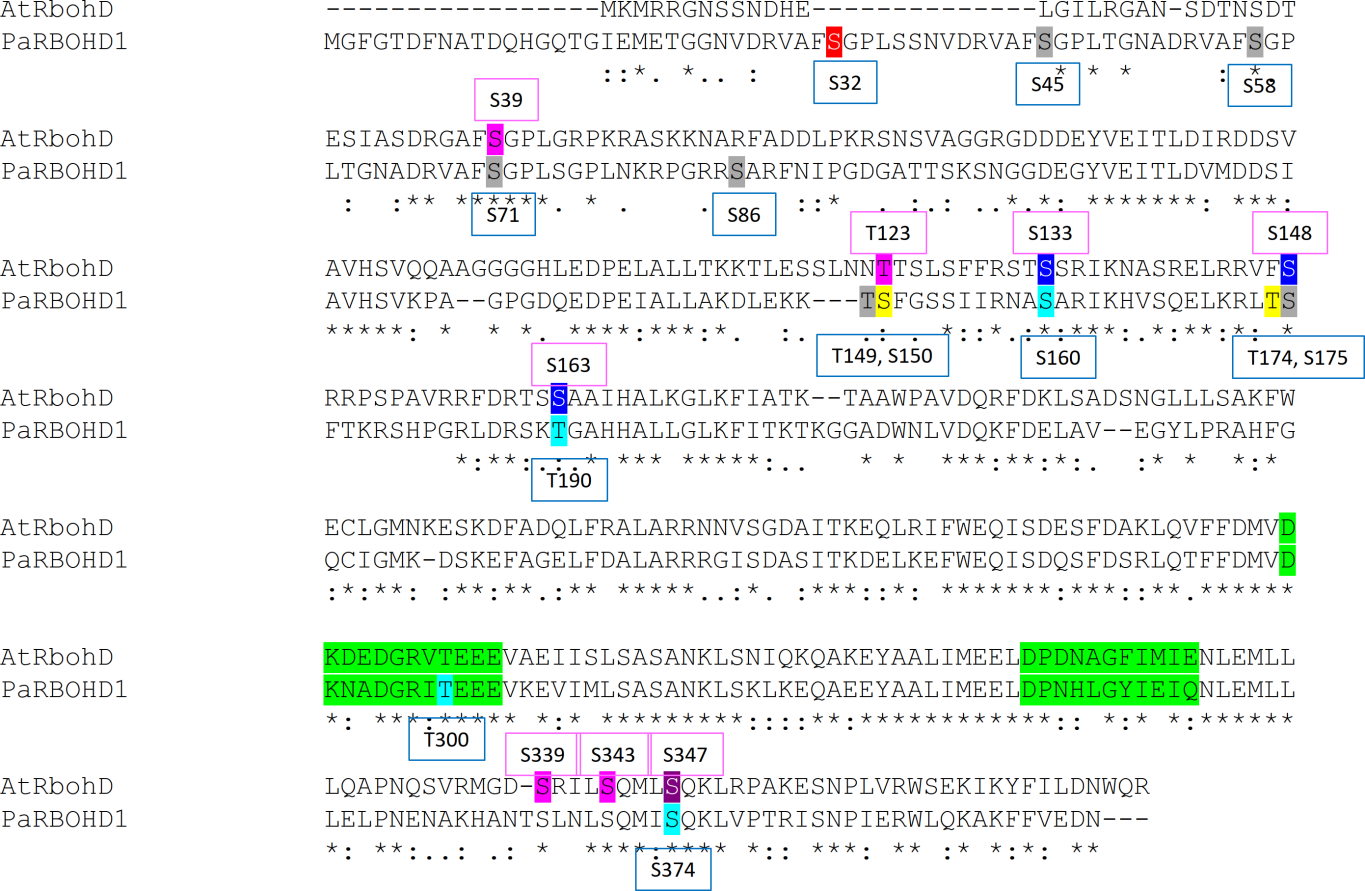
Phosphotarget sites in the N-terminus important for the PaRBOH1 activity, and comparison for those reported in AtRBOHD. The putative EF hands have been marked with a green colour. Colour codes in the AtRBOHD sequence: pink, a target site phosphorylated by BIK1; blue, a target site phosphorylated by CDKs; purple, a target site for both BIK1 and CDKs. Colour codes in the PaRBOH1 sequence: light blue, a phosphotarget site showing positive response in HEK cells; grey, a site showing no response in HEK cells; yellow, an amino acid having phosphorylation in the phosphoproteomic analysis and giving a positive response in HEK cell assays; red, an amino acid having phosphorylation in the phosphoproteomic analysis, but not giving a positive response in HEK cells. For references, see discussion.

We have shown for the first time in gymnosperms and tree species that PaRBOH1 has the ROS-producing activity activated by both Ca^2+^ and phosphorylation (**Figure 3**). Indeed, the cell extract of the developing xylem showed protein kinase activity phosphorylating specific serine and threonine residues of PaRBOH1 (**Table 1**). Alike in other plant species’ RBOHs, PaRBOH1 contains two EF-hand motifs in the cytosolic N-terminal region with a high sequence conservation to those present in AtRBOHs (**Figure 5**). Ca^2+^ binding to the EF-hand motifs has been shown to induce a conformational change that activates ROS production (Ogasawara et al., 2008; Oda et al., 2010). The results obtained with the Norway spruce PaRBOH1 in the present work support the idea that the synergistic activation by Ca^2+^ binding to the EF hand motifs and phosphorylation of several serine and threonine residues by protein kinases is a common regulatory mechanism conserved in seed plants (Ogasawara et al., 2008; Oda et al., 2010; Kaya et al., 2019).

*In silico* analysis followed by *in vitro* kinase assays were utilized to find out putative phosphorylation sites in the N-terminus of PaRBOH1 (**Table 1**). Next, a full-length *PaRBOH1* was transfected to HEK cells with each putative phosphotarget site individually mutated to alanine or aspartate, and the quantitative measurement of the ROS-producing activity conducted (**Figure 4**). These analyses combining the phosphoproteomic studies, *in vitro* phosphorylation, the quantitative assay of the ROS-producing activity and the site-directed mutagenesis showed that several serine and threonine residues seemed to be regulatory sites for the PaRBOH1 activity.

The phosphoproteomic analysis of the PaRBOH1 N-terminus after *in vitro* kinase assays using spruce soluble and membrane-bound kinases showed that S32, corresponding to S39 in AtRBOHD, and the following corresponding serine residues in the repetitive motif, *i.e.*, S45/58 and S71 were phosphorylated by both types of spruce endogenous kinases (**Tables 1** and **2**; **Supplementary Data S1**). In AtRBOHD, the site S39 is phosphorylated by BIK1 in the pathogen response in a Ca^2+^-independent way (Li et al., 2014; Kadota et al., 2014). Since non-elicited spruce trees were used as origins of developing xylem, phosphorylation of these serine residues may have a regulatory role during normal development. None of these serine residues in the repetitive motif of PaRBOH1 (**Figure 2C**, **Supplementary Table S3B**), however, gave consistent responses in the HEK cell assays (**Table 1**; **Figure 4**). The lack of a reproducible and consistent response for the repetitive sequence serine residues in the HEK cell assays suggests that these serine residues may be phosphorylated by some specific protein kinases in spruce but cannot be phosphorylated by endogenous protein kinases in the HEK293T cells.

The phosphoproteomic analysis indicated that T174, which gave a positive response in the HEK cell assays (**Figure 4**), is a novel site not yet recognized in other species (**Figure 5**). In some putative phosphorylation sites, the phosphoproteomic analysis showed no phosphorylation in a few positive sites of the *in vitro* kinase peptide assays and/or of the HEK cell assays (S160, T190, T300, S374), or showed phosphorylation when HEK cell assays did not give a consistent response (S32, T149, S175; **Table 1**). Out of these, T300 locates in the first EF-hand motif (**Figure 5**). The assays showed that the phosphomimic mutant of T300 has elevated ROS production after ionomycin-Ca^2+^ injection (**Table 1**), which suggests a beneficial effect of a prior T300 phosphorylation for the Ca^2+^ binding to the EF-hand and the resulting increase in PaRBOH1 activity. T190 was chosen for the HEK cell assays even if it did not give a positive response in *in vitro* kinase peptide assays, since it corresponds to the S163 site in AtRBOHD that has been shown to be a regulatory site (Benschop et al., 2007). S374 corresponds to S347 in AtRBOHD that is a phosphotarget site for both CDKs and BIK1 (Dubiella et al., 2013; Li et al., 2014; Kadota et al., 2014). It should be noted that the peptide TSLNL SQMIS QKLVP used in the *in vitro* kinase peptide assays with S374 as its 10^th^ amino acid also contains another serine at its 6^th^ position. This serine is S370 in the PaRBOH1 sequence, and corresponds to S343 in AtRBOHD, which is a verified phosphotarget site for the BIK1 (Kadota et al., 2014). Although S370 was not mutated for the HEK cell assays, it is possible that it serves as a phosphotarget site also in HEK cells, and in developing xylem of Norway spruce.

In AtRBOHD, S8, S9, S39, T123, S148, S152, S163, S343 and S347 are phosphorylated in response to pathogen-associated molecular patterns (PAMPs; Benschop et al., 2007; Nühse et al., 2007; Dubiella et al., 2013; Kadota et al., 2014; Zhang et al., 2018; Li et al., 2021). BIK1, for example, phosphorylates S39, T123, S339 and S343 and S347 of AtRBOHD (Li et al., 2014; Kadota et al., 2014), and CPKs phosphorylate AtRBOHD at S133, S148, S163 and S347 (Dubiella et al., 2013; Kadota et al., 2014). A receptor-kinase for extracellular ATP, DORN1, in turn, phosphorylates S22 and T24 in AtRBOHD in *in vitro* and *in vivo*, however, phosphorylation of S22, and not of T24, is essential for the ATP-triggered ROS burst (Chen et al., 2017). It is possible that in spruce, similar to Arabidopsis, a pathogen attack activates novel type(s) of kinases (e.g. an ortholog of a BIK1), that are not active during developmental lignification, leading to phosphorylation of sites not detected now in the proteomic analysis.

The presence of the four-times repeated motif in PaRBOH1 containing sites recognized by the BIK1 suggests that the serine residues in these motifs have an important function at certain physiological conditions. In several RBOHs (e.g. in AtRBOHD), the motif is present only once (**Figure 5**; **Supplementary Table S3A**). Interestingly, however, loblolly pine (*Pinus taeda*) contains a RBOH (PITA 000006695-RA) that shares a high homology to PaRBOH1 (91.5% identity) and has a very similar repetitive motif repeated for five times in its N-terminus (**Figure 2C**; **Supplementary Table S3B**). In addition, grape (*Vitis vinifera*) RBOHB (VV14G08690) has the core SPGL motif repeated for four times in row with slight variations in the sequence (data not shown). Significance of these repetitive sequences of the putative phosphorylation sites observed in several plant species is an interesting topic to be studied in future studies.

In the HEK cell assays, the ROS levels produced by PaRBOH1 were relatively low (**Figure 3**). Different AtRBOHs are known to vary in the ROS-producing activity by 100-times in the HEK cell system (Kaya et al., 2019). In general, the PaRBOH1-formed ROS levels were comparable to those of AtRBOHA, -D and -E.

Some studies suggest an important role of nitric oxide (NO) in regulating ROS production and scavenging (Gupta et al., 2020). Indeed, S-nitrosylation of C890, consisting of a nitrosothiol (SNO) formation through the addition of a NO moiety to a thiol group of a cysteine residue, down-regulates the RBOHD activity by comprising the oxidative burst induced by pathogen and the hypersensitive response associated cell death (Yun et al., 2011). Additionally, persulfidation of RBOHD cysteine residues by covalent addition of thiol groups at C825 and C890 enhanced ROS production in Arabidopsis (Shen et al., 2020). On the other side, NO-based post-translational modifications have been shown to modulate proteins involved in ROS scavenging, such as superoxide dismutase and ascorbate peroxidase, by S-nitrosylation and tyrosine nitration (Yang et al., 2015; Kolbert and Feigl, 2017). Out of the two predicted nitrosylation sites in PaRBOH1 sequence (**Supplementary Table S4**), first one is in the transmembrane area (Cys456) and the latter one close to the C-terminus (C933), approximately in the same area as the reported inhibitory nitrosylation event on C890 of RBOHD and therefore it deserves further investigation as a potential negative switch of RBOH activity in conifers.

This is the first study related to regulation of RBOH activity in gymnosperms including coniferous species. The data obtained indicate that regulation of RBOH activity by Ca^2+^ and phosphorylation is conserved in both gymnosperms and angiosperms including conifers, monocot and eudicot species. We have identified many putative phosphorylation sites by both soluble and membrane-bound protein kinases in the developing xylem. Identification of the responsible protein kinases expressed in the developing xylem would be an important and challenging future project. Transcriptomic data now available enable searches for candidate kinases among the similarly expressed genes. Once putative protein kinases are identified, co-expression of the putative kinases with PaRBOH1 in the heterologous expression system described in the present study would be useful to characterize the molecular regulatory mechanisms of PaRBOH1 by various protein kinases as well as phosphatases. In order to resolve the physiological significance of the PaRBOH1-mediated ROS production in Norway spruce, transgenic approaches are needed.

## Data availability statement

The PaRBOH1 coding sequence has been submitted to a NCBI GenBank with an accession number HQ592777.2. The proteomic dataset generated for this study are included in the article / Supplementary Material. The raw data of HDME LCMS runs, the PLGS 3.0 analysis result files, and the protein database used in the searches in ProteomeXchange (identifier PXD035518) are provided via Massive Spectrometry Interactive Virtual Environment (MassIVE Dataset): http://massive.ucsd.edu/ProteoSAFe/status.jsp?task=a3bd84481aa74996be9278bf1084c858".

## Author contributions

KN, AG, KHa, TL, HH, THT, KK and AK conceived the research and designed the experiments; KN, AG, KHa, TL, EV, TP, RS, TK, KHi, OB, SJ-L and AK performed the experiments; KN, AG, KHa, TL, EV, TP, RS, KHi, SJ-L and AK analyzed the data; KVF, HT and GW contributed materials/data/analysis tools; KN, AG, KHa, EV, TP, KK and AK wrote the manuscript with contributions of all authors. All authors contributed to the article and approved the submitted version.

## Funding

This work was supported by Academy of Finland (AK; decisions 251390, 256174, 283245 and 334184, SJ-L; decisions 331172 and 335972), Viikki Doctoral Programme in Molecular Biosciences (TL, EV), Integrative Life Science Doctoral Program (TL, EV), Jenny and Antti Wihuri Foundation (EV), MEXT, Japan (KK; KAKENHI grant numbers 50211884 and 25114515), JSPS, Japan (KK; KAKENHI grant number 15H01239), and Tokyo University of Science International Exchange Program (KK). Proteomic analyses were performed at the Meilahti Clinical Proteomics Core Facility, HiLIFE, supported by Biocenter Finland.

## Acknowledgments

We thank T. Warinowski and M. Huovila for cloning *PaRBOH1* cDNA.

## Conflict of interest

The authors declare that the research was conducted in the absence of any commercial or financial relationships that could be construed as a potential conflict of interest.

## Publisher’s note

All claims expressed in this article are solely those of the authors and do not necessarily represent those of their affiliated organizations, or those of the publisher, the editors and the reviewers. Any product that may be evaluated in this article, or claim that may be made by its manufacturer, is not guaranteed or endorsed by the publisher.
