# Supplementary material for: Regulation of PaRBOH1-mediated ROS production in Norway spruce by Ca2+ binding and phosphorylation
Source: Front Plant Sci. 2022 Oct 13;13:978586. doi: 10.3389/fpls.2022.978586 (PMC9608432; doi:10.3389/fpls.2022.978586)
Supplement: Supplementary file 2 [file Presentation_1.pdf]

Regulation of PaRBOH1-mediated ROS production in Norway spruce by Ca^{2+} binding and phosphorylation

Kaloian Nickolov, Adrien Gauthier, Kenji Hashimoto, Teresa Laitinen, Enni Väisänen, Tanja Paasela, Rabah Soliymani, Takamitsu Kurusu, Kristiina Himanen, Olga Blokhina, Kurt V. Fagerstedt, Soile Jokipii-Lukkari, Hannele Tuominen, Hely Häggman, Gunnar Wingsle; Teemu H. Teeri, Kazuyuki Kuchitsu, Anna Kärkönen

Supplementary information

Figure S1. A Coomassie-stained SDS-PAGE gel showing the Ni-NTA-column-purified N-terminus of PaRBOH1 after *in vitro* kinase assay.

Figures S2-S8. Screenshots from PLGS 3.0 analysis of a set of identified phosphopeptides.

Figure S9. A Western blot of mutated *PaRBOH1* constructs.

Table S1. Primers used for cloning the full-length Norway spruce *PaRBOH1* cDNA.

Table S2. Primers used for generating point mutations in the putative phosphotarget sites of PaRBOH1.

Table S3. (A) Sequences covering the (up-to-three) SGPL-like motif(s) used for WebLogo generation of the BIK-1 target site(s) corresponding to S39 in AtRBOHD in available plant RBOHs. (B) Repetitive motif sequences present in the N-terminus of PaRBOH1 and its closest *Pinus taeda* homolog.

Table S4. Summary of predictions for post-translational modifications in the PaRBOH1 sequence by Musite and DeepNitro online tools.

Data S1. Phosphoproteomic analysis: Identification of PaRBOH1 N-terminus and its tryptic peptides by LC-MS after *in vitro* kinase reactions using soluble cytoplasmic extract or microsomal membrane fraction of developing xylem of Norway spruce as sources for kinases.

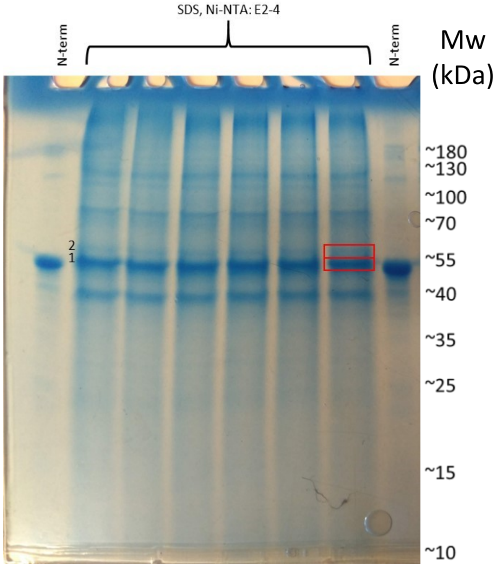

Figure S1. A Coomassie-stained SDS-PAGE gel showing the Ni-NTA-column-purified N-terminus of PaRBOH1 after *in vitro* kinase assay. Spruce microsomal membrane fraction was used as a source of kinases (see Materials and Methods for details). Bands 1 and 2 were cut out for phosphorylation site determination. Lines in the left and right contain the N-terminus not exposed to the *in vitro* kinase treatment (N-term). These were combined and used as a control in the phosphoprotein analysis.

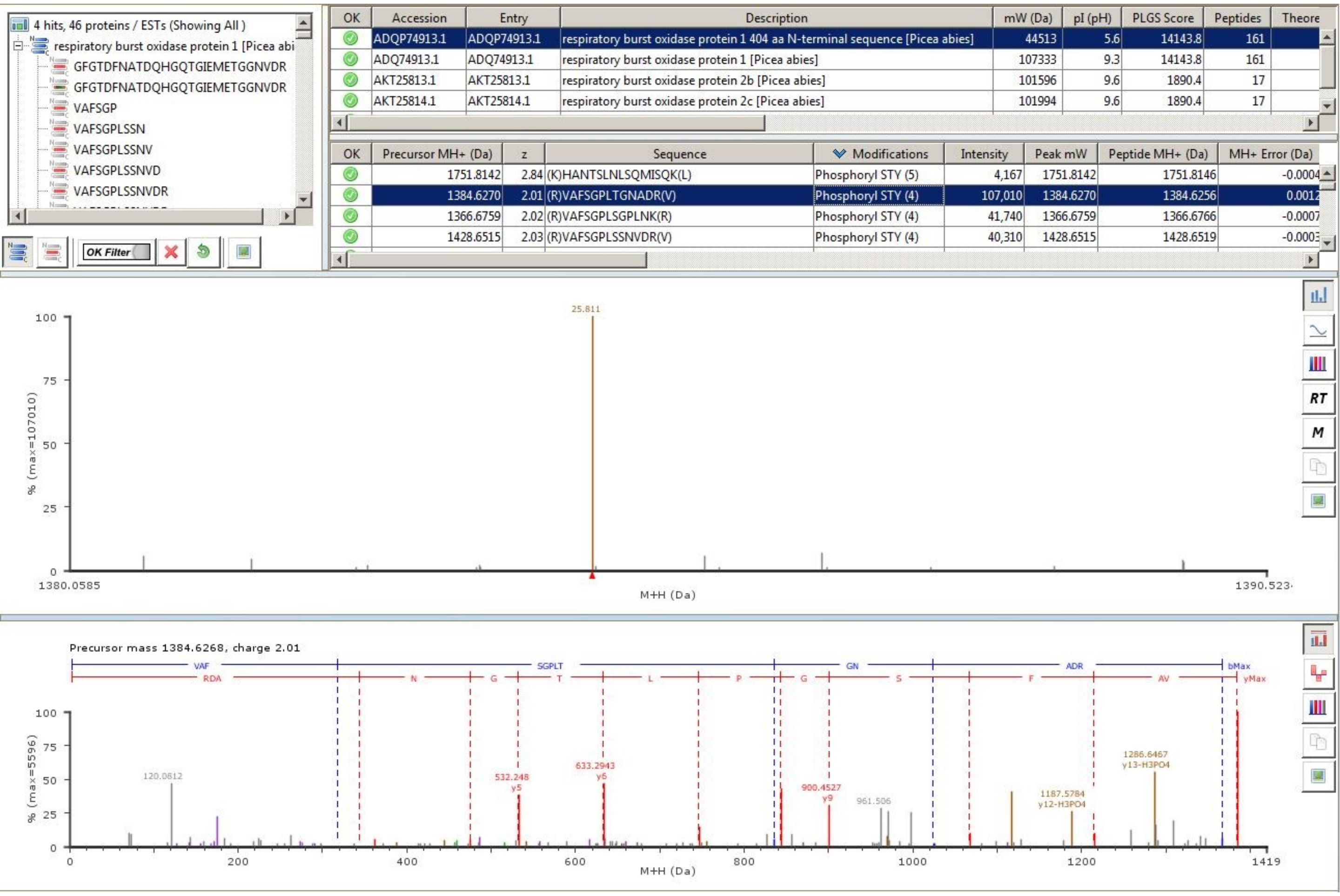

Figure S2. Parboh1_mb-kinase_band1_pep-VAFSGPLTGNADR

PLGS analysis viewing details of the phosphorylated peptide VAFpSGPLTGNADR in PaRBOH1 with the membrane kinase. Are shown among other details: peptide sequence, deconvoluted molecular mass ([M+H]$^+$, phosphorylation position, peak intensities, and MSMS spectrum.

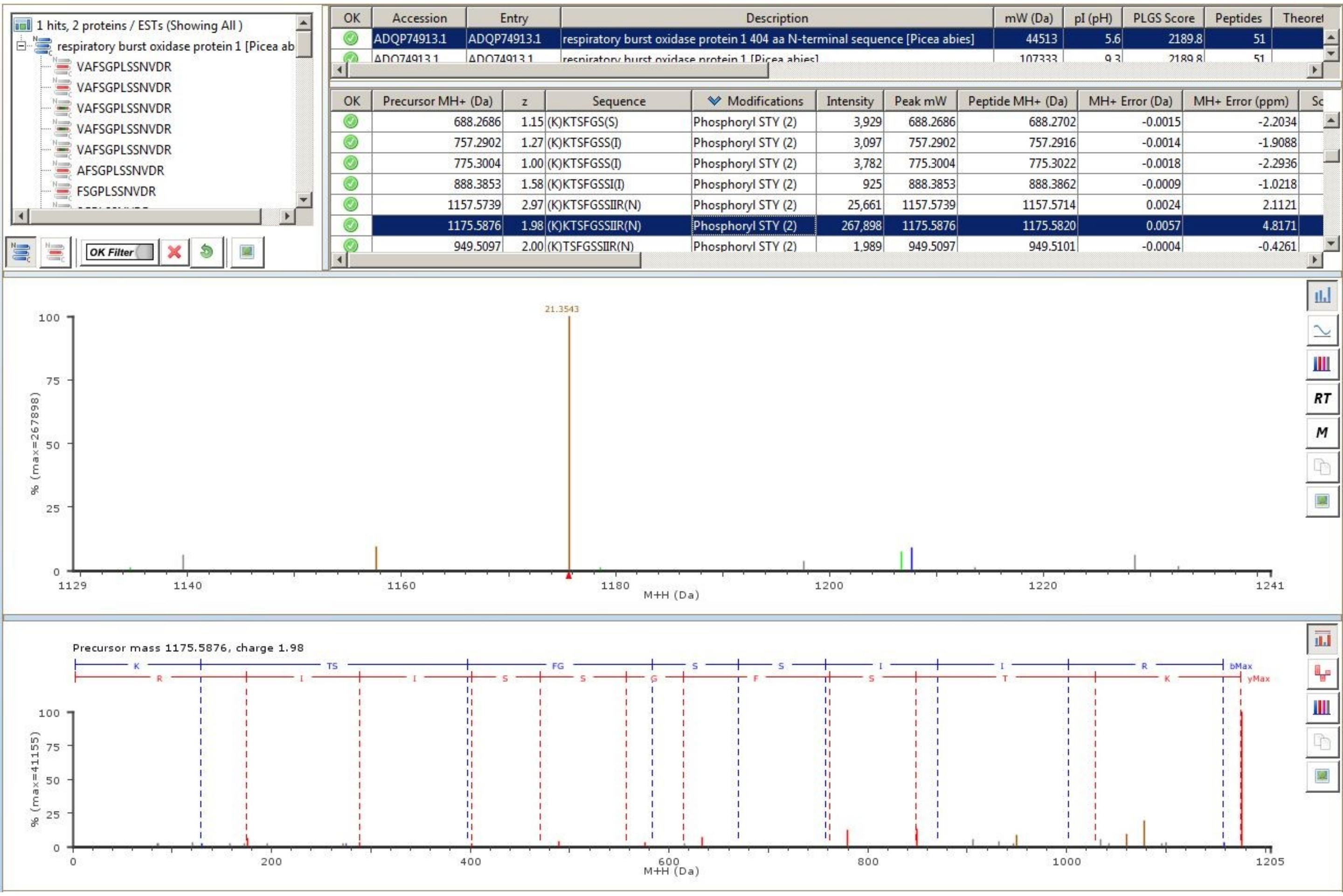

Figure S3. Parboh1_mb-kinase_band1_TiO2_eluate_pep-KTSFGSSIIR
PLGS analysis viewing details of the phosphorylated peptide KpTSFGSSIIR in PaRBOH1 with the membrane kinase, TiO_2 - enriched. Are shown among other details: peptide sequence, deconvoluted molecular mass ($[M+H]^+$, phosphorylation position, peak intensities, and MSMS spectrum.

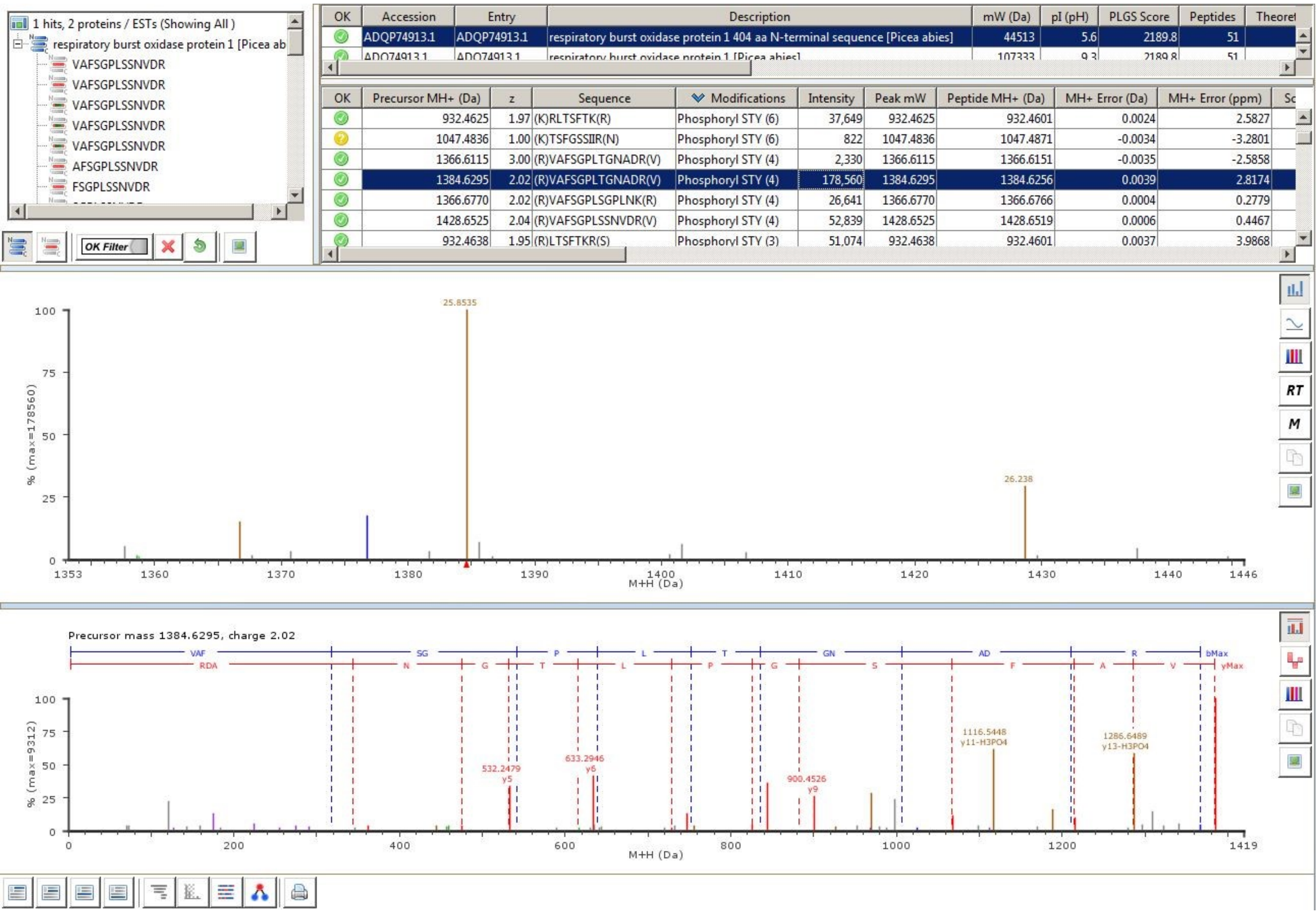

Figure S4. Parboh1_mb-kinase_band1_TiO2_eluate_pep-VAFSGPLTGNADR

PLGS analysis viewing details of the phosphorylated peptide VAFpSGPLTGNADR in PaRBOH1 with the membrane kinase, TiO_2 - enriched. Are shown among other details: peptide sequence, deconvoluted molecular mass ([M+H]$^+$, phosphorylation position, peak intensities, and MSMS spectrum.

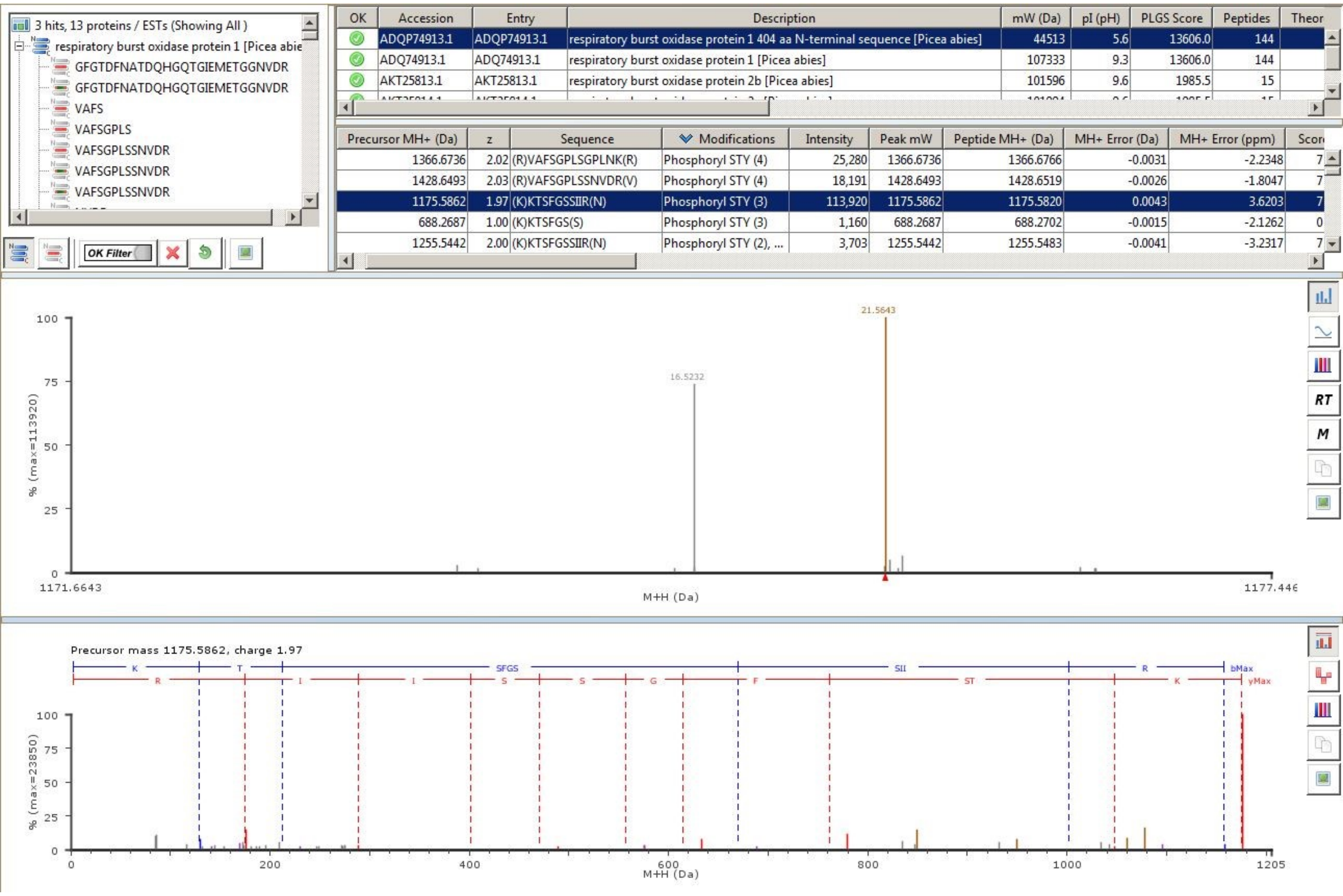

Figure S5. Parboh1_soluble-kinase_pep-KTSFGSSIIR

PLGS analysis viewing details of the phosphorylated peptide KTpSFGSSIIR in PaRBOH1 with the soluble kinase. Are shown among other details: peptide sequence, deconvoluted molecular mass ([M+H]$^+$, phosphorylation position, peak intensities, and MSMS spectrum.

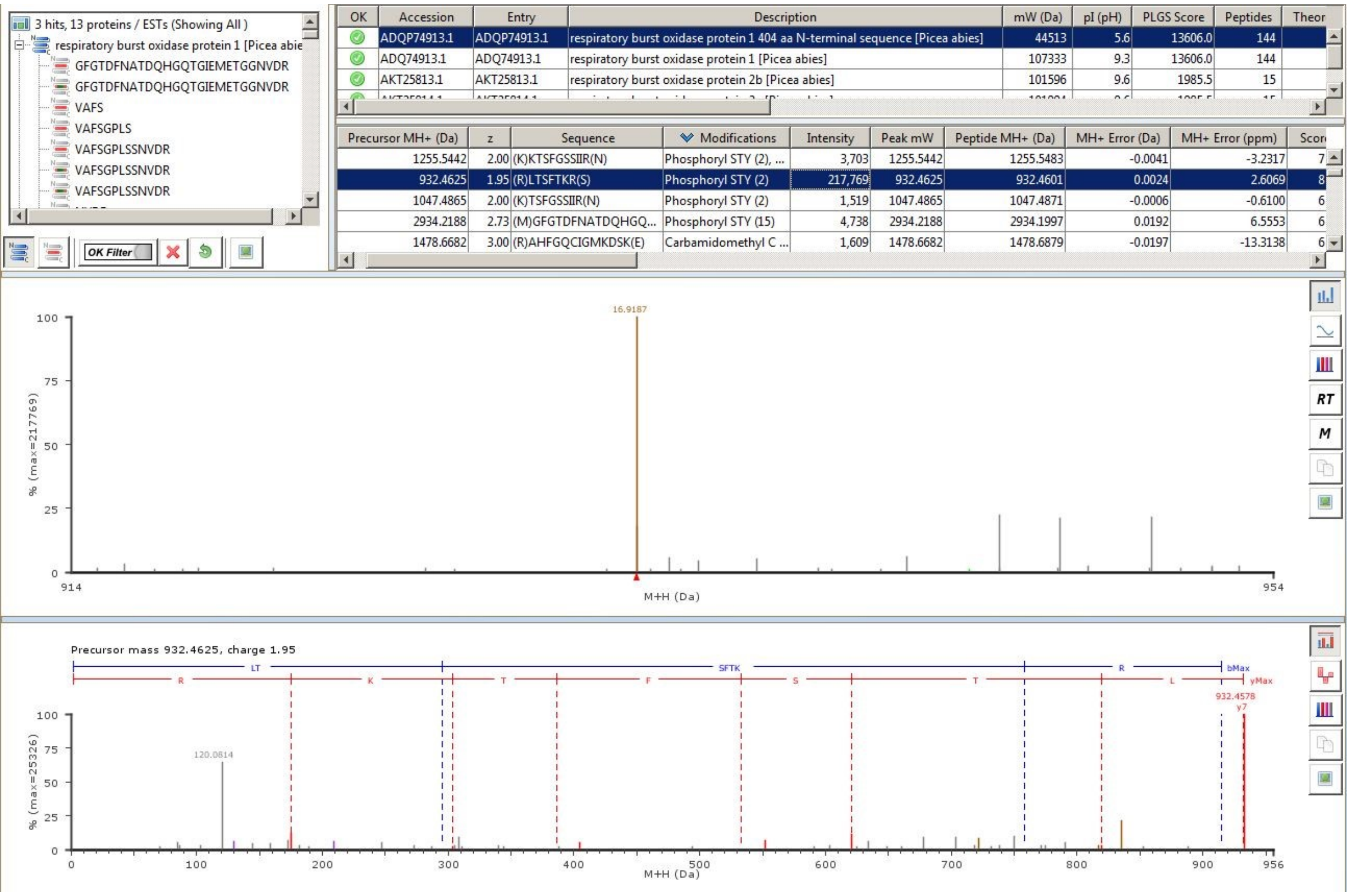

Figure S6. Parboh1_soluble-kinase_pep-LTSFTKR

PLGS analysis viewing details of the phosphorylated peptide LpTSFTKR in PaRBOH1 with the soluble kinase. Are shown among other details: peptide sequence, deconvoluted molecular mass ([M+H]$^+$, phosphorylation position, peak intensities, and MSMS spectrum.

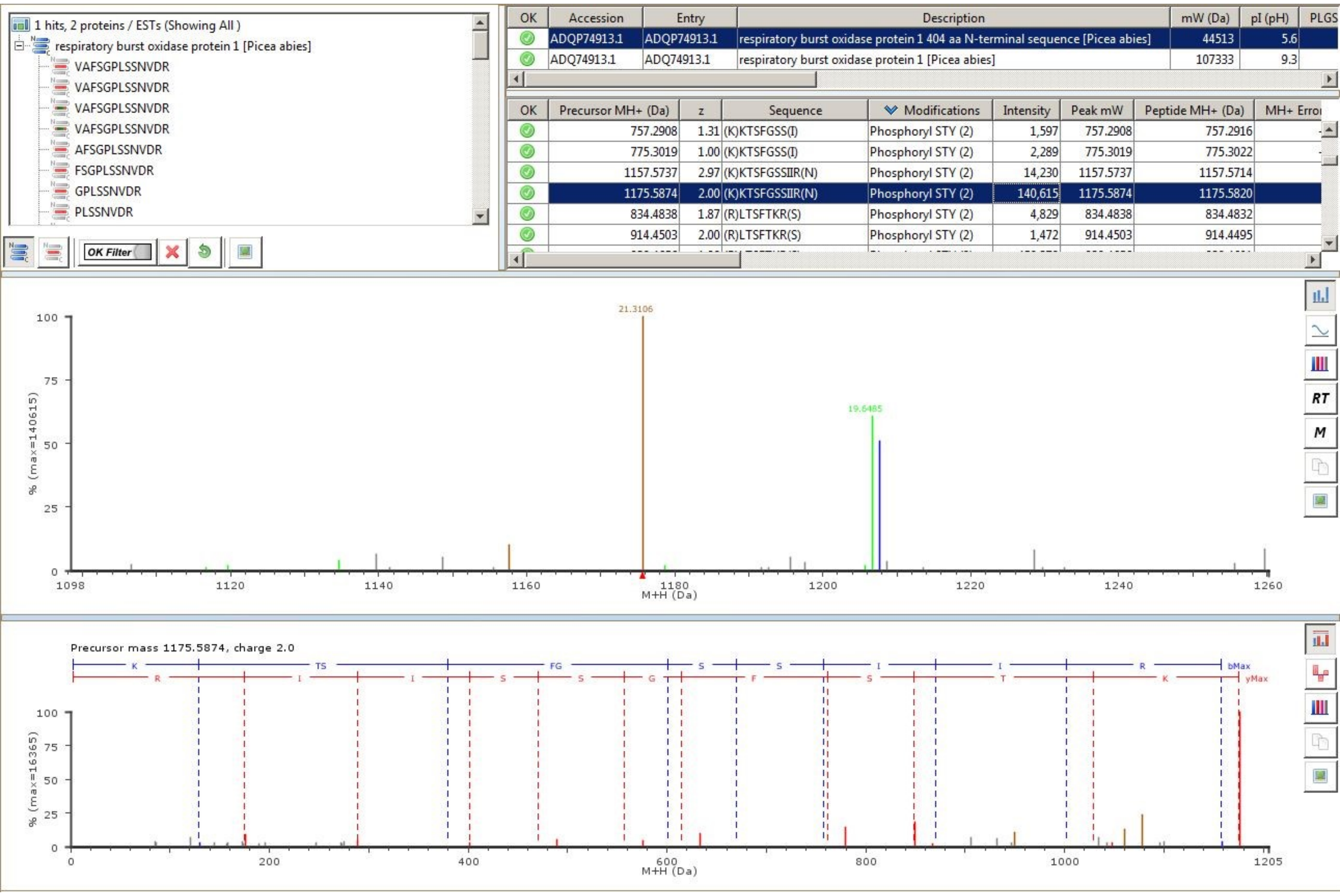

Figure S7. Parboh1_soluble-kinase_TiO2_eluate_pep-KTSFGSSIIR

PLGS analysis viewing details of the phosphorylated peptide KpTSFGSSIIR in PaRBOH1 with the soluble kinase, TiO_2 - enriched. Are shown among other details: peptide sequence, deconvoluted molecular mass ([M+H]$^+$, phosphorylation position, peak intensities, and MSMS spectrum.

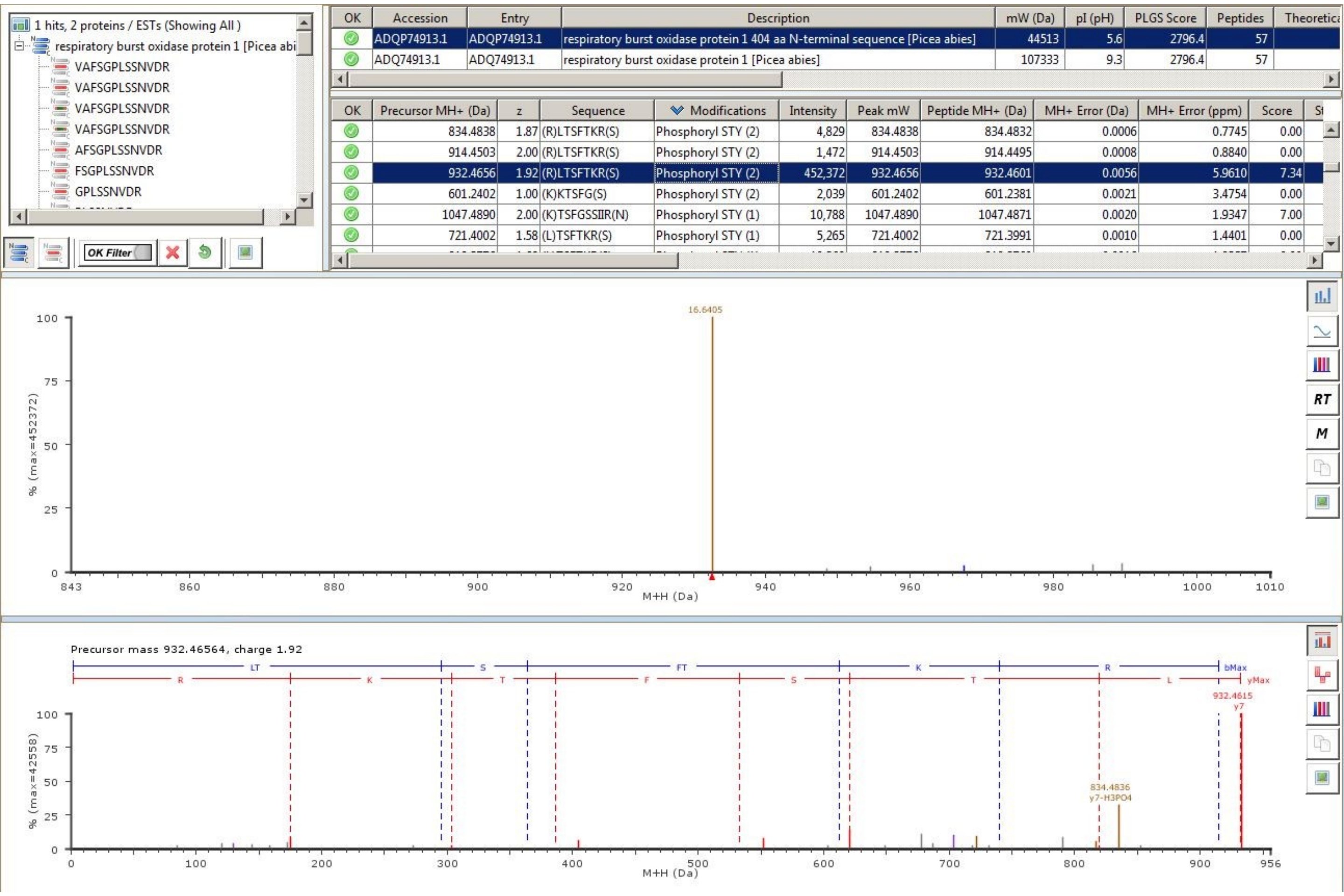

Figure S8. Parboh1_soluble-kinase_TiO2_eluate_pep-LTSFTKR

PLGS analysis viewing details of the phosphorylated peptide LpTSFTKR in PaRBOH1 with the soluble kinase, TiO_2 - enriched. Are shown among other details: peptide sequence, deconvoluted molecular mass ($[M+H]^+$), phosphorylation position, peak intensities, and MSMS spectrum.

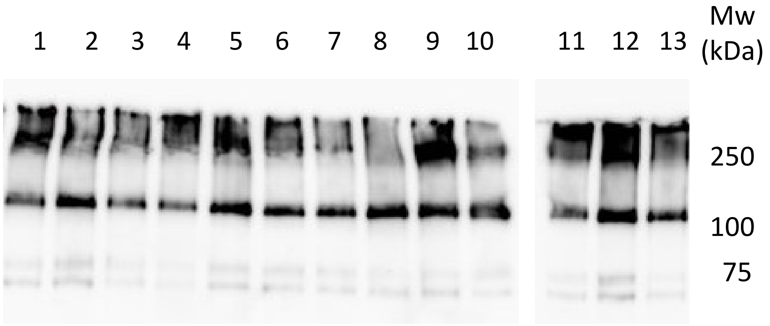

Figure S9. A Westerm blot of mutated PaRBOH1 constructs. Mutations of the predicted phosphotarget sites did not change the PaRBOH1 protein amounts produced in the HEK cells transfected with mutated/wild-type *PaRBOH1* constructs as revealed by a Western blot using an antibody against the FLAG-tag. Results from one experimental HEK cell assay on a multiwell plate are shown. Constructs: 1. T149A, 2. S160A, 3. T149D, 4. S160D, 5. S86A, 6. T174A, 7. S86D, 8. T174D, 9. S150A, 10. S175A, 11. S150D, 12. S175D, 13. wild-type *PaRBOH1*.

Table S1. Primers used for cloning the full-length Norway spruce *PaRBOH1* cDNA. The first PCR primers were designed using published RBOH sequences as a source of sequence information. N= any base; R = A or G; Y= C or T. [a]Locates in the 3'UTR.

PCR:	Sequence	Product
Forward:	5'- TGG TAY AAY AAR ACN ACN TGG ATG TAC -3'	
Reverse:	5'- AA NGA NCC YTG YTC YCT NGT NAC CCA -3'	Partial *PaRBOH1*
**3'RACE:**		
Forward:	5'- GGTAAAAGTGGTCTATTGAGAGCAGATTACATGCAAGGG -3'	3' end of *PaRBOH1*
Reverse:	Universal primers of the kit	
**5'RACE:**		
RbohUK1f:	5'- CCATTAAAACTTCGGTGTACTGGAGG -3'	
RBOH5full3r[a]:	5'- CCCAAAGCCTCGAACATATTCGCACTGG -3'	Full-length cDNA

Table S2. Primers used for generating point mutations in the putative phosphotarget sites of *PaRBOH1.* Serine (S) or threonine (T) were mutated to alanine (A, kinase-inactive) or to aspartate (D, phosphomimic). The number indicates the position of the mutated amino acid residue. F: forward, R: reverse.

Primer name	Sequence
S32A_F	5' - GATAGAGTTGCCTTCGCAGGACCTCTGAGCAGT - 3'
S32D_F	5' - GATAGAGTTGCCTTCGATGGACCTCTGAGCAGT - 3'
S32_R	5' - CACATTTCCACCAGTTTCCATCTCAATTCCTGTCTGACCGTG - 3'
S45A_F	5' - GCAGTAATGTGGATAGGGTTGCCTTCGCAGGACCTCTGACTGGT - 3'
S45D_F	5' - GCAGTAATGTGGATAGGGTTGCCTTCGATGGACCTCTGACTGGT - 3'
S45_R	5' - TCAGAGGTCCACTGAAGGCAACTCTATCCACATTTCC - 3'
S58A_F	5' - GACAGGGTTGCCTTCGCAGGACCTCTGACTGGTAATGC - 3'
S58D_F	5' - GACAGGGTTGCCTTCGATGGACCTCTGACTGGTAATGC - 3'
S58_R	5' - CGCATTACCAGTCAGAGGTCCACTGAAGGCAACCC - 3'
S71A_F	5' - GACAGGGTTGCCTTCGCAGGACCTCTGAGTGGCCC - 3'
S71D_F	5' - GACAGGGTTGCCTTCGATGGACCTCTGAGTGGCCC - 3'
S71_R	5' - CGCATTACCAGTCAGAGGTCCGCTGAAGGCAACCC - 3'
S86A_F	5' - AGAGGCCAGGCAGGAGGGCTGCCAGGTTTAATATACC - 3'
S86A_R	5' - GGTATATTAAACCTGGCAGCCCTCCTGCCTGGCCTCT - 3'
S86D_F	5' - AGAGGCCAGGCAGGAGGGATGCCAGGTTTAATATACC - 3'
S86D_R	5' - GGTATATTAAACCTGGCATCCCTCCTGCCTGGCCTCT - 3'
T149A_F	5' - GAAAGATCTGGAGAAGAAGGCTTCATTTGGGTCATCCAT - 3'
T149A_R	5' - ATGGATGACCCAAATGAAGCCTTCTTCTCCAGATCTTTC - 3'
T149D_F	5' - GGCGAAAGATCTGGAGAAGAAGGATTCATTTGGGTCATCCATAATT - 3'
T149D_R	5' - AATTATGGATGACCCAAATGAATCCTTCTTCTCCAGATCTTTCGCC - 3'
S150A_F	5' - GAGAAGAAGACTGCATTTGGGTCATCC - 3'
S150A_R	5' - CAGATCTTTCGCCAATAAAGCTATCTCT - 3'
S150D_F	5' - GGCGAAAGATCTGGAGAAGAAGACTGATTTTGGGTCATCCATAATTAGAAATG - 3'
S150D_R	5' - CATTTCTAATTATGGATGACCCAAAATCAGTCTTCTTCTCCAGATCTTTCGCC - 3'
S160A_F	5' - CATCCATAATTAGAAATGCTGCTGCAAGGATCAAGCATGTG - 3'
S160A_R	5' - CACATGCTTGATCCTTGCAGCAGCATTTCTAATTATGGATG - 3'
S160D_F	5' - TCATTTGGGTCATCCATAATTAGAAATGCTGATGCAAGGATCAAGCATGTG - 3'
S160D_R	5' - CACATGCTTGATCCTTGCATCAGCATTTCTAATTATGGATGACCCAAATGA - 3'
T174A_F	5' - ACAAGAACTCAAACGCCTTGCATCATTCACCAAGAGATC - 3'
T174A_R	5' - GATCTCTTGGTGAATGATGCAAGGCGTTTGAGTTCTTGT - 3'
T174D_F	5' - GTGTCACAAGAACTCAAACGCCTTGATTCATTCACCAAGAGATCCCA - 3'
T174D_R	5' - TGGGATCTCTTGGTGAATGAATCAAGGCGTTTGAGTTCTTGTGACAC - 3'
S175A_F	5' - AAACGCCTTACAGCATTCACCAAGAGA - 3'
S175A_R	5' - GAGTTCTTGTGACACATGCTTGATCCTT - 3'
S175D_F	5' - ACAAGAACTCAAACGCCTTACAGATTTCACCAAGAGATCCCATCCTG - 3'
S175D_R	5' - CAGGATGGGATCTCTTGGTGAAATCTGTAAGGCGTTTGAGTTCTTGT - 3'
T190A_F	5' - GATAGAAGTAAAGCAGGAGCCCATCA - 3'
T190D_F	5' - GATAGAAGTAAAGATGGAGCCCATCATG - 3'
T190_R	5' - CAGCCTTCCAGGATGGGATCTCT - 3'
T300A_F	5' - GATGGCCGAATCGCAGAAGAAGAAGT - 3'
T300A_R	5' - GGCATTCTTGTCGACCATGTCAAAAAATG - 3'

T300D_F	5' - GAATGCCGATGGCCGAATCGATGAAGAAGAAGTCAAAGAG - 3'
T300D_R	5' - CTCTTTGACTTCTTCTTCATCGATTCGGCCATCGGCATTC - 3'
S374A_F	5' - AGCCAAATGATAGCACAGAAGCTGGT - 3'
S374A_R	5' - CAAATTAAGGCTGGTGTTGGCATGTTT - 3'
S374D_F	5' - ACCAGCCTTAATTTGAGCCAAATGATAGACCAGAAGCTGGTGC - 3'
S374D_R	5' - GCACCAGCTTCTGGTCTATCATTTGGCTCAAATTAAGGCTGGT - 3'

Table S3. (**A**) Sequences covering the (up-to-three) SGPL-like motif(s) in available plant RBOHs used for WebLogo generation of the BIK-1 target site(s) corresponding to S39 in AtRBOHD. (**B**) Repetitive motif sequences present in the N-terminus of PaRBOH1 and its closest *Pinus taeda* homolog (PITA-000006695-RA).

A

SGPL-motif	RBOH (gene ID and annotation)
DRVAFSGPLSGPL----NKR	*Picea abies* MA_107643g0010 (HQ592777.2; PaRBOH1, last repeat only)
	Pinus taeda PITA_000006695-RA (last repeat only)
PVIAFSGHLGGALTGSSARK	*Picea abies* MA_12253g0010 (KT192592-KT192594; PaRBOH2)
PVIAFSGHLGSALSGSSARK	*Pinus taeda* PITA_000085516-RA
ERLAYSGPLSGPL----NKR	*Populus trichocarpa* potri.012G111600
ERVAYSGPLSGPL----NKR	*Populus trichocarpa* potri.015G109800
TRIGYSGPLSGPLVTT-TKK	*Populus trichocarpa* potri.005G026200
DRTAFSGPLASGGGGLLNKK	*Populus trichocarpa* potri.001G070900
DRTAFSGPLG-GGGGALNKK	*Populus trichocarpa* potri.003G159800)
DKASYCGPLGGPP----DKK	*Vitis vinifera* VV01G01490 (VvRBOHD)
DRRAFSGPISSTT---KPRK	*Citrullus colocynthis* ACF05504.2 (C1IHR0, CcRBOHD)
DRVGFSGPLSGPLSGPLNKR	*Amborella trichopoda* scaffold00016.300 (XP_006844712.1, RBOHC)
DRIGFSGPLSGPLSGPLNKR	*Lilia regale* ASV46345.1 (A0A248SMK2)
DRGAFSGPLGRP------KR	*Arabidopsis thaliana* AT5G47910 (AtRBOHD)
AEAGNSGPMSGGQLPPIYKK	*Arabidopsis thaliana* AT5G51060 (AtRBOHC)
DRASYSGPLSGPL----NKR	*Nicotiana tabacum* NP001313052.1 (NtRBOHC)
	Nicotiana tabacum CAC84140.1 (NtRBOHD)
	Solanum lycopersicum AF148534.1 (SlRBOHB)
	Solanum tuberosum Q2HXL0.2 (StRBOHC)
SRVGFSGSLV------SGKK	*Solanum tuberosum* Q948T9.1 (StRBOHB)
DRTSYSGPLSGPL-----KR	*Salvia splendens* TEY80299.1 (A0A4D9BEY4)
DKASFSGPLSGPL-----KR	*Salvia splendens* TEY20981.1 (A0A4D8YH87)
	Salvia splendens TEY16645.1 (A0A4D8YAQ3)
DKISFSGPLSGPL----NKR	*Salvia splendens* TEY39172.1 (A0A4D8ZXK4)
DRTAFSGPLGGPP---LNKK	*Manihot esculenta* ADR70882.1 (MeRBOHD)
--------MSGPL----NKK	*Manihot esculenta* ADR70892.1 (MeRBOHC)
RRAGFSGPLAT------NKR	*Manihot esculenta* ADR70881.1 (MeRBOHB)
ERAPYSGPLSGPL----NKR	*Manihot esculenta* ADR70880.1 (MeRBOHA)
RLVPHSGPLS--------KR	*Oryza sativa* OS11G33120 (OsRBOHI)
TLIPNSGNLGSS------NR	*Oryza sativa* OS01G25820 (OsRBOHB)
RVIPHSGTLS--------KK	*Zea mays* ZM02G42940 (A0A1D6F6A5)
	Zea mays ZM02G42960 (A0A1D6F6C5)
TLIPHSGNLGGS-----SRK	*Zea mays* ZM03G12260 (A0A1D6MT17)

RVIPHSGPLS--------KK	*Zea mays* ZM04G30500 (NP_001157759, A0A1D6QI90, ZmRBOHD)
SSGYMSGPMSGQLPPVY-KK	*Brassica napus* AVY54491.1 (BnRBOHC-1.1)
	Brassica napus AVY54492.1 (BnRBOHC-2.1)
DRSAFSGPLGRP------KR	*Brassica napus* AVY54494.1 (BnRBOHD-2.1)
	Brassica napus AVY54493.1 (BnRBOHD-1.1)

B

N-terminal stretch with repetitive motifs	RBOH
<u>DR</u>VAFSGPLSSNV<u>DR</u>VAFSGPLTGNA<u>DR</u>VAFSGPLTGNA<u>DR</u>VAFSGPLSGPL<u>NK</u>R	*Picea abies* MA_107643g0010 (HQ592777.2; PaRBOH1)
<u>DR</u>VAFSGPLSSNM<u>DR</u>VAFSGPLSGQG<u>DR</u>VAFSGPLGGSS<u>DR</u>VAFSGPLGGSA<u>DR</u>VAFSGPLSGPL<u>NK</u>R	*Pinus taeda* PITA_000006695-RA

Table S4. Summary of predictions for post-translational modifications in PaRBOH1 sequence by Musite and DeepNitro online tools. Combined output from the online Musite and DeepNitro (DN) algorithms; included are only residues with prediction scores above Musite general default cutoff level (0.5) as well as DeepNitro default medium cutoff levels (0.575 for W residues, 0.37 for C residues, 0.295 for Y residues).

Position in PaRBOH1	Post-Translation Modification	PTMscores
S5	Phosphoserine	0.832
S8	Phosphoserine	0.816
S13	Phosphoserine	0.548
S32	Phosphoserine	0.671
S33	Phosphoserine	0.829
S38	Phosphoserine	0.732
S42	Phosphoserine	0.509
S106	Phosphoserine	0.705
S110	Phosphoserine	0.66
S114	Phosphoserine	0.541
S116	Phosphoserine	0.69
S134	Phosphoserine	0.89
S145	Phosphoserine	0.764
S149	Phosphoserine	0.531
S151	Phosphoserine	0.594
S183	Phosphoserine	0.795
W208	Tryptophan Nitration (DN)	0.919
K291	Ubiquitination	0.599
S311	Phosphoserine	0.507
K314	Ubiquitination	0.523
K317	Ubiquitination	0.549
W401	Tryptophan Nitration (DN)	0.679
C456	S-Nitrosylation (DN)	0.375
S511	Phosphoserine	0.508
Y521	Tyrosine Nitration (DN)	0.407
K526	Ubiquitination	0.503
Y529	Tyrosine Nitration (DN)	0.518

W606	Tryptophan Nitration (DN)	0.768
W612	Tryptophan Nitration (DN)	0.804
K672	Ubiquitination	0.634
S739	Phosphoserine	0.542
S795	Phosphoserine	0.701
S797	Phosphoserine	0.6
S799	Phosphoserine	0.501
S802	Phosphoserine	0.819
T804	Phosphothreonine	0.5
S806	Phosphoserine	0.875
T817	Phosphothreonine	0.571
S818	Phosphoserine	0.892
K847	Ubiquitination	0.582
W912	Tryptophan Nitration (DN)	0.641
C933	S-Nitrosylation (DN)	0.459
S946	Phosphoserine	0.606
K965	Ubiquitination	0.502
